# Bioresorbable Materials on the Rise: From Electronic Components and Physical Sensors to In Vivo Monitoring Systems

**DOI:** 10.1002/advs.201902872

**Published:** 2020-01-19

**Authors:** Antonino A. La Mattina, Stefano Mariani, Giuseppe Barillaro

**Affiliations:** ^1^ Dipartimento di Ingegneria dell'Informazione Università di Pisa Via G. Caruso 16 56122 Pisa Italy

**Keywords:** biodegradable sensors, bioresorbable materials, implanted devices, material dissolution, transient electronics

## Abstract

Over the last decade, scientists have dreamed about the development of a bioresorbable technology that exploits a new class of electrical, optical, and sensing components able to operate in physiological conditions for a prescribed time and then disappear, being made of materials that fully dissolve in vivo with biologically benign byproducts upon external stimulation. The final goal is to engineer these components into transient implantable systems that directly interact with organs, tissues, and biofluids in real‐time, retrieve clinical parameters, and provide therapeutic actions tailored to the disease and patient clinical evolution, and then biodegrade without the need for device‐retrieving surgery that may cause tissue lesion or infection. Here, the major results achieved in bioresorbable technology are critically reviewed, with a bottom‐up approach that starts from a rational analysis of dissolution chemistry and kinetics, and biocompatibility of bioresorbable materials, then moves to in vivo performance and stability of electrical and optical bioresorbable components, and eventually focuses on the integration of such components into bioresorbable systems for clinically relevant applications. Finally, the technology readiness levels (TRLs) achieved for the different bioresorbable devices and systems are assessed, hence the open challenges are analyzed and future directions for advancing the technology are envisaged.

## Graphical abstract

Bioresorbable materials that fully dissolve in the body with biologically benign byproducts provide a unique opportunity to engineer new electrical, optical, and sensing components into implantable biomedical systems with direct access to organs, tissues, and biofluids, without need of secondary device‐retrieving surgery. Herein, the current picture and future direction of bioresorbable devices and systems are presented, with specific attention to challenges that still need to be addressed toward real‐word applications.

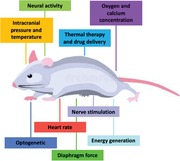

## 1 Introduction

Bioresorbable materials that fully dissolve in the body with biologically benign byproducts provide a unique opportunity to engineer new electrical, optical, and sensing components into implantable biodegradable systems that eliminate any boundary with the human body, granting direct access to organs, tissues, and biofluids without the need of secondary device‐retrieving surgery that may cause tissue lesion or infection.^1^

Traditional medical devices designed to be implanted in the human body to treat either acute or chronic diseases (e.g., cardiac pacemakers, cochlear implants, coronary stents, articular prostheses) are made of (or coated with) inert biocompatible materials and optimized to warrant the organ functionality from days to years, depending of the targeted problem/disease, before surgical retrieval/replacement (when needed).^2^, ^3^ While for some specific cases (e.g., heart stimulation, hearing loss, coronary heart disease) implanted devices ideally working/operating over the whole patient life are required, in many other cases (e.g., muscle stimulation, bone growth stimulation, neuro stimulation, wound healing) the devices are required to work/operate only for a prescribed amount of time, so that surgical retrieval is eventually needed, which could be potentially dangerous for patient health.^4^

Bioresorbable medical devices designed to be implanted in the human body and operate for a prescribed time, before they undergo spontaneous dissolution in patient body, would therefore represent a new paradigm in patient care.

Besides, the continuous diffusion of noninvasive and minimally invasive sensor systems for personalized medicine and sport&wellness activities envisages an ever‐growing electronic waste that poses serious environmental hazards, given the pervasive presence and rapid turn‐over of electronic devices in everyday life.^5^, ^6^, ^7^, ^8^, ^9^ The development of biodegradable electronic components and systems that facilitate disposal by natural dissolution in ambient or mild conditions without environmental threats could mitigate electronic waste problems.^10^, ^11^, ^12^

Bioresorbable components and biodegradable electronics would greatly benefit one from each other findings, both pursuing the same goal of developing high‐quality devices that operate for a prescribed time and then vanish under well‐defined conditions. In both cases, mechanisms for triggering and tuning the dissolution of these devices by using different physico‐chemical conditions, such as, acidic^13^, ^14^ or basic^15^, ^16^ solutions, light exposure,^17^ and low^18^ or high temperatures,^19^, ^20^ are envisaged to provide a more accurate on‐demand control over dissolution.

Summarizing, an ideal bioresorbable system is required to monitor physio‐pathological parameters of clinical interest (e.g., intracranial pressure), transmit the acquired data to an external receiver, and produce a feedback action on the relevant organs/tissues aimed at improving the clinical status of the patient. If implanted, the materials that compose the system are required to be biocompatible and degradable in physiological conditions, further resulting in biocompatible degradation byproducts. Eventually, the system operation and performance are required to be stable until programmed (or even better, externally triggered) dissolution occurs, to ensure correct monitoring and effective caring of the patient throughout the healing process, and the degradation products need to be rapidly resorbed and/or drained by the patient organism to avoid the risk of foreign body reactions both at local and systemic levels, especially for a chronic implant. For instance, imagine an infection sentinel system being implanted in a surgical site, able to continuously monitor bacterial charge, releasing antibiotics and sending alerts to physicians in case of infection risk, and that after complete surgical wound healing totally and safely dissolves within days. It would be a groundbreaking achievement for patient life quality and health safety.

**Figure 1:**
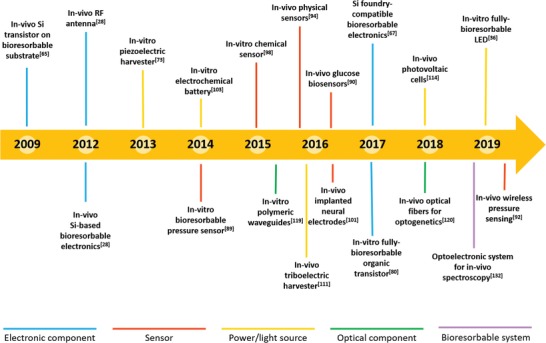
Milestones on bioresorbable devices and systems. Milestones achieved over the last decade on bioresorbable devices and systems for in vitro and in vivo applications, highlighting the impressive outcomes of the research field in the last few years.

In spite of the huge research effort paid over the latest years on this subject, many important issues remain open and have still to be addressed toward the achievement of such a bioresorbable medical system, including lifetime of electronic and optical components, stability and lifetime of power sources, on‐demand triggering of material dissolution, development of chemical sensors operating in vivo, and long‐term material biocompatibility in vivo. A summary of the milestones achieved over the last decade on this field is given in **Figure** **1**.

In this Review, differently from recent reviews on transient, degradable, and bioresorbable materials and devices mainly focusing on material preparation and fabrication processes,^21^, ^22^, ^23^, ^24^, ^25^, ^26^, ^27^ we will focus on functional operation and physiological dissolution of bioresorbable devices and systems, aimed at assessing the TRL achieved by the different facets of the bioresorbable technology. We will first discuss the dissolution chemistry and kinetics, as well as the biocompatibility of the main bioresorbable materials, including inorganic semiconductors, oxides, and metals, as well as organic polymers, systematically grouping material class and dissolution rate. Next, we will review the state‐of‐the‐art bioresorbable devices and systems, including electronic components, sensors and actuators, power sources, and optical devices. Eventually, we will draw the current picture and envisage future direction on bioresorbable devices and systems, with specific attention to the challenges that still need to be overcome toward real‐word applications.

## 2 Bioresorbable Materials and Dissolution Chemistry

**Figure 2:**
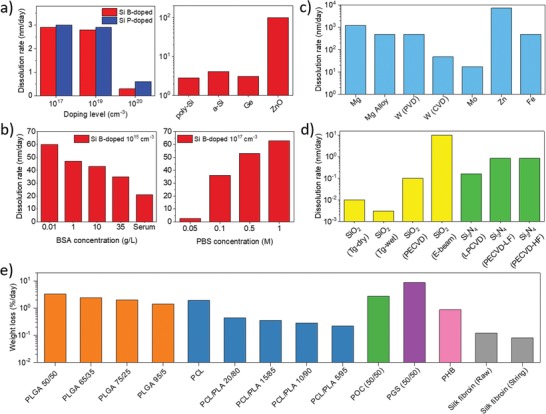
Dissolution rates of the principal bioresorbable materials in physiological conditions. Summary of the dissolution rates of the different materials used in bioresorbable devices and systems at pH 7.4 and 37 °C, namely, a,b) semiconductors,^30^, ^31^, ^33^, ^35^, ^36^ c) metals,^43^ d) oxides and nitrides,^39^ and e) polymers.^52^, ^53^, ^54^, ^55^, ^56^, ^57^, ^58^

In this section we will review dissolution chemistry and kinetics, and biocompatibility (when available) of the main bioresorbable materials both in vitro and in vivo, namely: inorganic semiconductors (e.g., silicon (Si), germanium (Ge), and zinc oxide (ZnO)), oxides and nitrides (e.g., silicon oxides and silicon nitrides), metals (e.g., magnesium (Mg), zinc (Zn), tungsten (W), and molybdenum (Mo)), and polymers and organic materials (e.g., polyesters, waxes, cyclic poly(phtalaldehyde) (cPPA), and silk). **Figure** **2** summarizes the etching rates of these materials in physiological conditions (i.e., pH 7.4 and 37 °C), with organic and inorganic semiconductors being mostly used as active part of bioresorbable transistors, metals mainly used as electrodes and for interconnections, polymers typically employed as supporting substrate and flexible coating, and oxides/nitrides being used as encapsulation layer.

### 2.1 Inorganic Semiconductors

Among the different bioresorbable materials, monocrystalline silicon in the form of nanomembranes (Si NMs) represents an emerging choice for the fabrication of high‐performance transient electronics and bioresorbable devices.^28^, ^29^

Hwang et al.^30^ studied the kinetics of hydrolysis of monocrystalline Si NMs with different dopants (type and concentration) in different solutions (buffers and biofluids), evaluating in vitro and in vivo cytotoxicity. The dissolution of single‐crystalline Si NMs in aqueous media is governed by Equation (1), namely

(1) $$\text{Si}+4{\text{H}}_{\text{2}}\text{O}\to \text{Si}{\left(\text{OH}\right)}_{4}\left(\text{aq}\right)+2{\text{H}}_{2}$$

Si NMs (B‐doped 10^16^ cm^−3^, 100 nm thick) were fully and uniformly dissolved in 24 h upon immersion in 1 m phosphate buffered saline (PBS) (at pH 7.4 and 37 °C) and in bovine serum (at pH ≈ 7.4 and 37 °C), with a comparable dissolution rate *r* (by definition, thickness of dissolved material per day) of ≈100 nm day^−1^. By changing type (P‐ and B‐doped) and concentration (10^17^ through 10^20^ cm^−3^) of dopants, the dissolution rate was found to be more sensitive to concentration rather than type of dopants, at least for 70 nm thick Si NMs in PBS (0.1 m, pH 7.4 and 37 °C). For instance, dopant concentrations of 10^17^ cm^−3^ and 10^19^ cm^−3^ resulted in *r* ≈ 3 nm day^−1^ regardless of the type of dopants, whereas at concentration of 10^20^ cm^−3^ the etching rate was ≈0.8 and ≈0.3 nm day^−1^ (roughly a factor 3) for P and B dopants, respectively. In vitro cytotoxicity tests were carried out on metastatic breast cancer cells (MDA‐MB‐231) cultured on Si NMs subjected to continuous dissolution for consecutive days. Although the Si NMs were fully dissolved in 4 days in the culture medium, the cell viability was excellent after ten days (93 ± 4%). In vivo studies were performed by subcutaneous implantation of Si NMs in mice dorsal skin. Biodegradability and biocompatibility (no cytotoxicity and no weight loss in mice) of the implant over 5 weeks were assessed using high‐density polyethylene (HDPE) as Food and Drug Administration (FDA)‐approved control material. After 5 weeks, no residues of degradation were visible at the implant sites by stereomicroscopic analysis, while immunohistochemistry of the skin sections and hematoxylin and eosin (H&E) staining proved similar levels of immune cells to those of HDPE (i.e., no cytotoxicity).

Expanded studies on dissolution kinetics of semiconductors for transient electronics were reported by Kang et al.^31^ The authors correlated the dissolution rates of 100 nm thick NMs of polycrystalline silicon (p‐Si), amorphous silicon (a‐Si), silicon–germanium alloy (SiGe), and germanium (Ge) in aqueous solutions with different pH (7–10) and temperature (room temperature and 37 °C) values. Equation (2) applies to germanium dissolution in aqueous media, namely

(2) $$\text{Ge}+{\text{O}}_{2}\left(\text{aq}\right)+{\text{H}}_{2}\text{O}\to {\text{H}}_{2}{\text{GeO}}_{3}\left(\text{aq}\right)$$

Dissolution rates at physiological temperature (37 °C) were higher than those at room temperature for all the tested materials, which is in agreement with the Arrhenius equation.^32^ The increase of pH significantly increases the dissolution rate of p‐Si, a‐Si, and Ge NMs. For instance, at physiological conditions (37 °C and pH 7.4) *r* was 2.8, 4.1, and 3.1 nm day^−1^, respectively, while at pH = 10 the NMs were completely dissolved in a few hours, regardless of the material. Conversely, the SiGe alloy (Si_8_Ge_2_ (100)) showed higher stability with pH than the other materials, and no significant dissolution was recorded until pH = 8 after 16 days, while only 25 nm were dissolved at pH = 10. Biocompatibility was evaluated by culturing two different cell types (L929 mouse fibroblast and whole splenocytes harvested from mouse spleen) over 72 h onto the NMs for cytotoxicity studies. Cell viability suggested the nontoxic nature of the four dissolved materials, compared to HDPE used as control.

Two years later, the same research group deepened the understanding on dissolution kinetics of Si NMs in ground‐water and biofluid media.^33^ The authors investigated the dissolution rate of Si NMs (B‐doped, 10^15^ cm^−3^, 200 nm thick) in PBS (1×) spiked with different concentrations of albumin (0.01–35 g L^−1^), Si(OH)_4_ (0–300 mg L^−1^) and cations (Na^+^, Mg^2+^ and Ca^2+^, 1 × 10^−3^ m) at 37 °C. The increase of the protein concentration slowed down the dissolution rate due to augmented protein adsorption onto the NM surface; moreover, regardless of the concentration (and presence) of proteins, the dissolution rate reduced by increasing the concentration of Si(OH)_4_, consistently with the chemical equilibrium reported in Equation (1). Conversely, the presence of cations in the aqueous medium (i.e., PBS at pH 7.4, with 35 g L^−1^ of proteins at 37 °C) led to an accelerated dissolution rate, which was greater for divalent cations (namely, Ca^2+^ and Mg^2+^) with respect to monovalent cations (Na^+^). For instance, the presence of 1 × 10^−3^ m Ca^2+^ increased the dissolution rates of a factor ≈1.5, from 35 nm day^−1^ (in absence of cations) to 51 nm day^−1^. This result was explained with the ability of divalent cations to deprotonate surface silanol groups, formed by absorption of water on Si surface, and to enhance, in turn, the reactivity of water and siloxane groups, as suggested for SiO_2_ dissolution.^34^ The Si NMs were eventually investigated as water barrier for encapsulation of bioresorbable electronics. Two different strategies were examined to decrease the dissolution rate in the presence of Ca^2+^: the use of heavily doped Si NMs (B‐doped, 10^20^ cm^−3^), for which a decrease of the dissolution rates of ≈40‐fold was achieved; and nanometric superficial oxidation with UV ozone (UVO, 3 nm thick) or O_2_ plasma (20 nm thick) of Si NMs, for which a delay of the Si dissolution of 10 and 30 days, respectively, was demonstrated.

Yin et al.^35^ further reported that in aqueous media the dissolution kinetics of Si NMs was affected by the presence of cations (e.g., Na^+^, Ca^2+^, and Mg^2+^) and anions (Cl−, HCO_3_−, HPO_4_^2^−) which are commonly available in biologic and environmental fluids at concentrations ranging from 0.1 to 50 g L^−1^. The study reported that the dissolution rate of Si NMs (B‐doped, 10^17^ cm^−3^) was ≈1 nm day^−1^ at low ionic concentrations (e.g., 0.05 m K_2_HPO_4_/KH_2_PO_4_), and increased up to 65 nm day^−1^ at high concentrations (e.g., 1 m K_2_HPO_4_/KH_2_PO_4_). Density functional theory (DFT) simulations showed that the presence of anions could weaken the interaction between Si atoms and nearby Si–Si backbones, thus accelerating the dissolution kinetics.

Dissolution of ZnO in physiological conditions was recently investigated by Lu et al.,^36^ who used ZnO as semiconductor in the fabrication of bioresorbable light‐emitting diodes (LEDs). ZnO samples (200 µm × 200 µm squares, thickness ≈200 nm) immersed in PBS at pH = 7.4 and 37 °C completely dissolved in 48 h, with a dissolution rate of about 4.1 nm h^−1^ (≈100 nm day^−1^). The Zn^2+^ concentration, measured at the end of the dissolution tests using a fluorescent chelating agent, namely, zinquin, was estimated to be 33 ng mL^−1^, confirming a full and direct degradation of ZnO according to hydrolysis reaction reported in Equation (3), namely

(3) $$\text{ZnO}+{\text{H}}_{\text{2}}\text{O}\to {\text{Zn}}^{2+}+2{\text{OH}}^{-}$$

without formation of insoluble precipitates of Zn(OH)_2_ (solubility ranging from 3.2–21 µg mL^−1^, experimentally observed at 25–50 °C, pH 7.4). Further, possible precipitates of Zn(OH)_2_, formed at concentrations exceeding Zn^2+^ solubility, could dissolve via formation of elementary Zn^2+^ ions and 2OH^−^ hydroxides according to the equilibrium reaction Zn(OH)_2_ ↔ Zn^2+^ + 2OH^−^ (with solubility constant *K*_ps_ = 3 × 10^−17^), driven by the continuous excretion of the dissolved ions (i.e., Zn^2+^) from the organism (Le Chatelier's principle).^37^ The biocompatibility of ZnO and, in turn, of its byproducts was assessed by monitoring the dissolution of ZnO nanostructures in horse blood and serum.^38^

### 2.2 Silicon Oxides and Nitrides

In the field of transient electronics, silicon oxides and nitrides have also key significance for digital and analog circuits and thin film displays, due to their use as gate and interlayer dielectrics, passivation coatings, and barriers against water penetration.^39^, ^40^

Kang et al.^39^ studied the hydrolysis kinetics of thin films of silicon oxides and nitrides in diverse aqueous solutions at different pH levels, ion concentrations, temperatures, and for different deposition methods. Silicon oxides dissolve in aqueous media according to Equation (4), namely

(4) $${\text{SiO}}_{2}+2{\text{H}}_{\text{2}}\text{O}\to \text{Si}{\left(\text{OH}\right)}_{4}\left(\text{aq}\right)$$

The reaction is initiated and kinetically influenced by OH− concentration (i.e., pH of solution). At this purpose, the authors investigated the dependence of dissolution rate on pH (i.e., range 7.4–12), temperature (i.e., room temperature and 37 °C), and type of oxide (i.e., thermal oxide, grown in O_2_ (tg‐dry) or H_2_O vapor (tg‐wet), and deposited oxide, obtained through plasma‐enhanced chemical vapor deposition (PECVD) and electron‐beam evaporation (E‐beam)). The dissolution rate increased with temperature (according to Arrhenius equation)^32^ and with pH for all the tested oxides. Once temperature and pH values were given, the different oxides were etched at different rates *r*_tg‐dry_ < *r*_tg‐wet_ < *r*_PECVD_ < *r*_E‐beam_. In particular, *r*_tg‐dry_ ranged from 6.3 × 10^−4^ to 1 nm day^−1^, *r*_tg‐wet_ from 4.7 × 10^−4^ to 1.4 nm day^−1^, *r*_PECVD_ from 2.2 × 10^−2^ to 14 nm day^−1^, and *r*_E‐beam_ from 3.5 to 282 nm day^−1^, at pH 7.4 and room temperature, and at pH 12 and 37 °C, respectively. The different etching rates were explained in terms of different physico‐chemical properties of oxides grown/deposited with different methods, namely: thermal oxide is uniformly dense and has a homogeneous stoichiometry; PECVD oxide has different densities and nonhomogeneous stoichiometry; E‐beam oxide can be affected by nanoscale fragmentation.

Stability of thermal silicon oxide as biofluid barrier was further investigated by Lee et al.,^40^ who examined the effect of various ionic species present in biofluids, revealing the dependence of the dissolution rate on both cation (Na^+^, K^+^, Mg^2+^, and Ca^2+^) and anion (Cl− and HPO_4_^2^−) concentrations, near neutral pH values. The authors found that the presence of Ca^2+^ accelerates the dissolution rate more effectively than Mg^2+^ and Na^+^, regardless of pH value. For instance, at pH 7.5 the dissolution rates were 34, 56, and 110 nm day^−1^ with 30 × 10^−3^ m NaCl, 10 × 10^−3^ m MgCl_2_, and 10 × 10^−3^ m CaCl_2_ solubilized in the aqueous solution, respectively. This was explained with the enhanced ability of Ca^2+^ in Si—O bonding polarization and, in turn, in making it more prone to hydrolysis. These findings were in agreement with former reports on the role of alkali metals in quartz dissolution.^41^ Further, the dissolution rate was higher in the presence of HPO_4_^2^− rather than Cl−: for instance, for 1 m ionic strength and pH = 6.8 the dissolution rates were 146 ± 9 and 64 ± 7 nm day^−1^, respectively. The anionic‐specific increase of dissolution rate with phosphate was explained in terms of strong hydrogen bonding with the silica surface.

Similar studies on dissolution kinetics were also carried out for silicon nitride.^39^ Silicon nitride dissolves in aqueous media in two chemical steps: 1) oxidation to silicon dioxide, according to Equation (5), namely

(5) $${\text{Si}}_{3}{\text{N}}_{4}+6{\text{H}}_{2}\text{O}\to 3{\text{SiO}}_{2}+4{\text{NH}}_{3}$$

and 2) hydrolysis of silicon oxide, according to Equation (4). The overall reaction is reported in Equation (6), namely

(6) $${\text{Si}}_{3}{\text{N}}_{4}+12{\text{H}}_{2}\text{O}\to 3\text{Si}{\left(\text{OH}\right)}_{4}\text{(aq) }+4{\text{NH}}_{3}$$

Being the silicon dioxide an intermediate byproduct of the reaction, the dissolution rate of silicon nitride also increased with temperature and pH, as previously reported for silicon dioxide. Two different silicon nitride deposition techniques were considered: low pressure chemical vapor deposition (LPCVD) and PECVD. The dissolution rate of LPCVD nitride resulted lower than that of PECVD nitride (at pH 7.4 and 37 °C dissolution rates were 0.16 and 0.83 nm day^−1^, respectively), due to better stoichiometry and higher density of the former. Stacked layers of SiO_2_ and Si_3_N_4_ were employed in the encapsulation of a magnesium serpentine resistor (300 nm thick) as a proof‐of‐concept application in transient electronics. In particular, it was shown that, by using a triple bilayer of PECVD‐deposited SiO_2_ and Si_3_N_4_ (single material layer thicknesses of 200, 200, and 100 nm, respectively, resulting in a total thickness about 1 µm) for the encapsulation, a lifetime of roughly 10 days was guaranteed for the resistor in deionized water (DIW) at room temperature.

The biocompatibility of SiO_2_ and Si_3_N_4_ was demonstrated by subcutaneous implantation of both the materials in rodents using a stainless steel wire mesh cage as carrier. Exudates were sampled after 4, 7, 14, and 21 days, and inflammatory response and leukocyte concentrations (leukocytes mL^−1^) were evaluated. The inflammatory response generated by SiO_2_ and Si_3_N_4_ was not significantly different from that produced by the empty cage used as control, monitored over the experiment duration.^42^

### 2.3 Metals

Metals are interesting in bioresorbable (and implantable) devices for their peculiar mechanical and electronic properties.^43^

Mg was successfully used for electrical contacts and interconnections in silicon‐based transient electronics by Hwang et al., due to its ease of processing, fast dissolution rate in aqueous media, and full biocompatibility.^28^ Yin et al.^43^ later reported on chemistry and mechanism of dissolution of Mg in deionized water. Mg dissolves in aqueous media according to Equation (7), namely

(7) $$\text{Mg}+2{\text{H}}_{2}\text{O}\to \text{Mg}{\left(\text{OH}\right)}_{2}+{\text{H}}_{2}$$

Although Mg dissolution macroscopically exhibited high uniformity, scanning electron microscope (SEM) analysis revealed that dissolution occurred with the formation of micropores and needle‐like structures. In addition, X‐ray photoelectron spectroscopy (XPS) analysis showed that the main product of the dissolution was Mg(OH)_2_, with MgO and MgCO_3_ as byproducts due to the presence of oxygen and CO_2_ in ambient atmosphere, respectively. The outer surface was mainly composed of a Mg(OH)_2_ layer that featured an increased thickness as the dissolution proceeded, according to experimental results carried out in DIW (*r* = 7.2 nm day^−1^) and Hanks's balanced saline solution (HBSS) (*r* = 115 nm day^−1^) as a simulated body fluid.

Other metals, including W, Mo, and Zn, showed similar dissolution chemistries and transient behaviors both in DIW and HBSS.^43^ Zn (comparably to Mg) showed a fast transient mechanism both in DIW (1.7 nm day^−1^) and in biofluids (7.2 nm day^−1^), while W and Mo showed a slower and better tunable degradation both in DIW and HBSS (in the range of 10^−2^ nm day^−1^ ). For this reason, the latter two materials should be preferred for medical devices that require metals to have direct contact with biological tissues, for instance, for physiological electrical signal sensing.

The biocompatibility of Mg and its alloys,^44^ Fe,^45^ and Zn^46^ was already largely assessed in vivo (with materials and devices implanted in animal models or humans), through evaluation of inflammatory response, and histological and immunofluorescence analysis. Mo and W, although of considerable interest for bioresorbable devices, have less comprehensive data on biocompatibility related to the concentration of byproducts released.^43^

All the discussed metals were deposited via vacuum‐based technologies, such as, chemical vapor deposition and sputtering. However, a huge interest is nowadays directed toward the development of new and low‐cost methods to reliably fabricate bioresorbable metal films, such as laser printing and metal nanoparticle‐filled conductive inks.^47^, ^48^, ^49^

### 2.4 Polymers and Organic Materials

Bioresorbable polymers are mostly employed as substrate support for degradable devices and systems.^50^ Compared to all the above‐discussed materials, polymeric materials offer higher flexibility in tuning the dissolution timescale. Indeed, polymer dissolution can be triggered by using external signals,^14^ and its rate can be tailored by varying molecular weight, material crystallinity, chemical structure, hydrophilic/hydrophobic character, and exploiting bulk/surface erosion mechanisms.^51^

Commonly used bioresorbable polymers are poly(lactide) (PLA/PLLA) and poly(lactide‐*co*‐glycolide) (PLGA),^52^ poly(caprolactone) (PCL),^53^, ^54^ poly(octanediol‐*co*‐citrate) (POC),^55^ poly(glycerol sebacate) (PGS),^56^ poly(hydroxybutyrate) (PHB),^57^ silk,^58^ and, recently reported, candelilla wax.^49^ All these polymers are polyesters (or polyamide, silk), and their depolymerization mechanism in aqueous solution is based on acid or basic hydrolysis of the ester (or amide) bond. As an example, Equation (8) reports the hydrolysis of PLGA


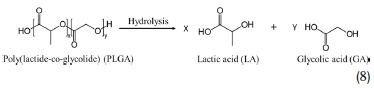


The dissolution rates (expressed as polymer mass% dissolved per day in PBS at pH 7.4 and 37 °C) ranged from a minimum value of 0.22% day^−1^ for a blend of PCL and PLA (5/95) to a maximum value of 8.6% day^−1^ for PGS copolymer (50/50).

The biocompatibility of these polymers was largely assessed, as reported in former scientific studies.^59^, ^60^, ^61^, ^62^, ^63^, ^64^ For instance, PLA and PLGA biocompatibility was demonstrated in vivo through histological and immunologic tests after implantation of microspheres in rats;^59^ PCL biocompatibility was assessed through evaluation of the viability of L929 mouse fibroblasts cultured on films of the polymer;^60^ biocompatibility of POC was assessed by monitoring form and phenotype of porcine chondrocytes cultured on POC scaffolds;^61^ PGS biocompatibility was demonstrated using human cardiac mesenchymal stem cells and rat cardiac progenitor cells cultured on PGS membrane through evaluation of viability after staining with DAPI;^62^ biocompatibility of PHB was assessed by subcutaneous implantation in rats and evaluation of the inflammatory response on tissue after 4 and 12 weeks;^63^ silk biocompatibility was widely demonstrated both in vitro and in vivo for applications in wound healing and in tissue engineering of bone, cartilage, tendon, and ligament tissues.^64^

Won et al.^49^ reported on the use of natural waxes as materials for long‐time and hydrophobic encapsulation in bio/ecoresorbable electronics. Soy, myrtle, and candelilla waxes, derived from soybeans (via soybean oil), *myrica cerifera* (myrtle), and candelilla shrubs, respectively, were tested. Waxes are known to be environmentally and biologically degradable (hydrolysis of ester and anhydride bonds in aqueous solution), conversely to their petroleum‐derived counterparts. Biodegradation tests carried out on candelilla wax (800 µm thick foil) subcutaneously implanted in the dorsal region of rats revealed a thickness reduction of roughly 28 µm after three months. Further, candelilla wax was used as biodegradable matrix for the preparation of conductive pastes, e.g., loaded with tungsten microparticles (C‐Wax, 800 µm thick foil). Biocompatibility of both candelilla and C‐wax were investigated through subcutaneous implantation in the abdominal region of mice for two months. No remarkable histological changes were recorded after staining of the skin tissue section with H&E (compared to sham surgery skin). In addition, no adverse immunological response was verified after staining with antibody CD45, a pan‐immune cell marker.

In 2014, Lopez Hernandez et al.^14^ reported an innovative photodegradable or photo‐triggerable transient electronic device using cPPA as substrate. cPPA is a metastable polymer that depolymerizes into monomers once triggered by acidic conditions. The authors developed a transient system based on a film of cPPA and a photoacid generator, namely, 2‐(4‐methoxystyryl)‐4,6‐bis(trichloromethyl)‐1,3,5‐triazine (MBTT), linked to the backbone of cPPA. When the device was exposed to UV light (379 nm), the MBTT generated a highly reactive Cl^•^ radical rapidly forming hydrochloric acid (HCl), that reacted with cPPA triggering its depolymerization. As a proof‐of‐concept, a free‐standing 2.5% MBTT/cPPA film featuring an array of transistors was fabricated. The MBTT/cPPA film was initially robust under ambient conditions (in air) but the continuous exposition to a UV lamp activated the depolymerization process, transforming the film in an oily and shapeless agglomerate. The whole degradation process lasted about 4 h, though degradation rates could be tuned by modifying the photoacid generator concentration and the irradiance of the UV source. No biocompatibility studies of the material, byproducts, or device were reported by the authors.

Compared to the previously reported bioresorbable polyester polymers, silk‐based natural peptide fibers (naturally produced by *Bombyx mori* larvae) have been reported as a more attractive alternative for the design of bioresorbable sensors and electronics because of the robust mechanical properties, the ability to tailor dissolution and biodegradation rates (from hours to years), the formation of noninflammatory amino acid degradation products, and the option to prepare the materials at ambient conditions to preserve sensitive electronic functions.^58^, ^65^, ^66^ From the chemical point of view, silk consists of two main proteins, namely, sericin and fibroin, the latter being the structural center of the silk, while the former being the sticky material surrounding it. In vitro dissolution tests of raw silk and of fibroin and sericin filaments (PBS at 37 °C) showed that dissolution occurred with weight losses of 0.12 and 0.08% day^−1^, respectively.^58^

## 3 Bioresorbable Electrical Devices

In this section we will review the main electrical components made out, either in total or partially, of bioresorbable materials, namely, active (e.g., transistors) and passive (e.g., resistors) components, sensors (e.g., physical and chemical), and power sources (e.g., batteries and energy harvesters), paying specific attention to their performance versus biodegradability and biocompatibility in vitro and/or in vivo, where reported.

From now on, for those components in which biodegradability and/or biocompatibility of some of the materials were not explicitly investigated with the component itself, the reader should refer to Section 2 of this Review.

### 3.1 Electrical Components

Several inorganic and organic bioresorbable materials have been used as active semiconductors, gate dielectrics, electrical interconnections, and supporting substrates for the fabrication of degradable electrical components, toward the achievement of more complex electronic circuits and (in perspective) small microprocessors.

#### 3.1.1 Inorganic Transistors

A great impulse to bioresorbable electronics came from the discovery that implanted silicon nanomembranes dissolve in vivo under physiological conditions,^28^ allowing the use of already mature silicon integrated circuit (IC) technology for the fabrication of transient electronic devices.^67^

In 2009, Kim et al.^65^ proposed silk as a bioresorbable substrate for silicon‐based n‐channel metal‐oxide‐semiconductor field effect transistors (MOSFETs). The transistors were fabricated leveraging silicon technology, i.e., silicon dioxide as dielectric and gold for electrical contacts deposited and patterned on crystalline silicon NMs (200 nm thick, p‐type) by using photolithography, reactive ion etching, plasma enhanced chemical vapor deposition, and electron beam evaporation. After fabrication, the transistors were transfer‐printed on a silk freestanding membrane using a poly(dimethylsiloxane) (PDMS) stamp. Silk was chosen as substrate because of its excellent biocompatibility and tunable dissolution, which can be tailored by modification of its chemical structure. Threshold voltage and electron mobility of the transistors were around 0.2 V and 500 cm^2^ V^−1^ s^−1^, respectively, which were comparable to those of traditional silicon MOSFETs. When immersed in water, the 25 µm thick silk substrate dissolved within 3 min, releasing the transistors in water. Electrical characteristics of the transistors after silk dissolution showed only small variations compared to as‐prepared transistors (threshold and electron mobility of 0.5 V and 440 cm^2^ V^−1^ s^−1^, respectively). In vivo studies were carried out by subcutaneously implanting the transistors laying on silk substrate in mice and retrieving them after 2 weeks. The results showed only partial dissolution of both silk film and transistors in this time frame, as well as the lack of any inflammation around the implant site. Although additional studies were required, these initial in vivo experiments suggested promising developments for this form of biodegradable electronics.

**Figure 3:**
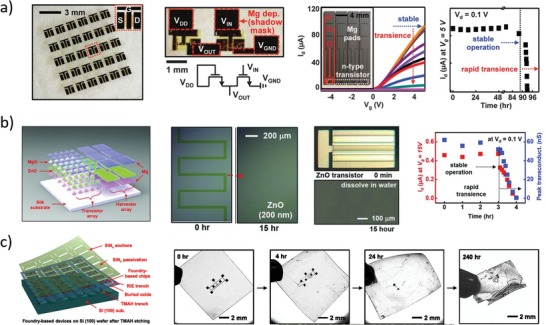
Representative examples of inorganic transistors. a) Left: Photographs of an array of completely bioresorbable silicon transistors on silk substrate.^28^ Middle‐left: Optical micrograph of a transient silicon nMOSFET inverter on silk substrate, and respective circuit scheme. Middle‐Right: Electrical characteristic of a nMOSFET during in vitro degradation experiment in deionized water at room temperature; inset: optical micrograph of the device configuration used for the degradation experiment. Right: Functional degradation of the silicon transistor. Reproduced with permission.^28^ Copyright 2012, American Association for the Advancement of Science. b) Left: Sketch of the ZnO‐based bioresorbable transistor array.^73^ Middle: Representative optical microscope images during dissolution experiments of a ZnO trace (left) and a complete transistor (right) immersed in deionized water at room temperature. Right: Functional degradation of a MgO‐encapsulated ZnO transistor during immersion in deionized water. Reproduced with permission.^73^ Copyright 2013, John Wiley and Sons. c) Left: Sketch of the foundry‐compatible bioresorbable Si transistor.^67^ Right: Representative photographs during device dissolution in buffer at pH 7.4 and 70 °C. Reproduced with permission.^67^ Copyright 2017, The Authors, Published by National Academy of Sciences.

In fact, three years later (2012) the same research group reported^28^ the groundbreaking invention of transient electronic devices made of silicon, allowing facile fabrication of bioresorbable electronic components (**Figure** **3**a). In particular, silicon nanomembrane‐based transistors were fabricated using silicon (300 nm thick, p‐type) as active material, SiO_2_ (100 nm) as gate dielectric, Mg (300–400 nm) for electrical contacts, MgO (400 nm) and silk (70 µm) as encapsulant layers. The disintegrable n‐channel MOSFETs exhibited threshold voltage and electron mobility of about 0.6 V and 500 cm^2^ V^−1^ s^−1^, respectively. In vitro dissolution in DIW at room temperature resulted in stable operation for about 4 days, followed by rapid performance degradation in 30 min (due to Mg electrode disintegration). NAND and NOR logic ports were fabricated and tested in similar ways, as well.

One year later, Hwang et al.^68^ reported on silicon‐based bioresorbable n‐type MOSFETs and nMOS inverters fabricated on a custom silicon on insulator (SOI) wafer. The transistors were fabricated with standard IC processes on a 100 nm thick p‐silicon layer, using SiO_2_ as gate and interlayer dielectrics, and Mg for electrical contacts. Additional SiO_2_ (100 nm, PECVD) and Si_3_N_4_ (400 nm, PECVD) layers were used as encapsulating and masking materials, respectively. Etching of the silicon on the back‐side was carried out in tetramethyl ammonium hydroxide (TMAH) to release the transistors, which were then transfer‐printed on a silk substrate, before removal of the Si_3_N_4_ layer. The fabricated bioresorbable nMOSFETs exhibited threshold voltage and electron mobility around 0.6 V and 530 cm^2^ V^−1^ s^−1^, respectively. In vitro experiments in PBS at pH 7.4 and 37 °C were performed to monitor both kinetics and mechanisms of the transistor dissolution. Magnesium was completely dissolved in 36 h, silicon dioxide in 2 weeks, and silicon in 4 weeks. Monitoring of the transistor performance in PBS at pH 7.4 and 37 °C was carried out using an additional magnesium dioxide layer (800 nm thick) for encapsulation. A stable operation was recorded for 8 h, followed by performance degradation in the subsequent 45 min. In vivo experiments were carried out with an array of transistors transfer‐printed on a silk substrate and subcutaneously implanted in mice. The results showed full transistor dissolution and no inflammation response after 2 weeks (verified through histological examination of the tissue surrounding the implant), while substrate dissolution was not yet complete. Using similar approaches, silicon transistors were successfully fabricated on different bioresorbable polymeric substrates, such as PLGA,^69^ poly(octanediol‐*co*‐citrate),^70^ levan polysaccharide,^71^ as well as on metal foils.^72^

In the same year, Dagdeviren et al.^73^ reported on a fully bioresorbable ZnO FET (Figure 3b). Magnesium oxide was used as gate dielectric, magnesium for electrical contacts, and zinc oxide as semiconductor. Silk fibroin was used both as substrate and as encapsulation layer. The threshold voltage of the transistors was 1 V, and carrier mobility was 0.95 cm^2^ V^−1^ s^−1^. Dissolution experiments were performed in deionized water at room temperature: the silk substrate immediately dissolved when immersed in water, while the other components slowly dissolved in 15 h without visible flaking or cracking. To demonstrate functionality in aqueous solution, the transistors were fabricated on glass substrate and encapsulated with magnesium oxide (500 nm thick), resulting in a stable operation over 3 h, followed by a rapid functional degeneration in the next 45 min. All the materials used in this work spontaneously dissolved in water, producing hydroxides (e.g., Mg(OH)_2_ and Zn(OH)_2_) or polypeptides that were environmentally and biologically benign (as discussed in Section 2).

More recently (2017), Chang et al.^67^ developed foundry‐compatible bioresorbable MOSFETs, inverters, and logic ports (Figure 3c). The transistors were fabricated on standard 6‐inch SOI wafers by an external foundry using common IC processes and materials, and then (in laboratory) they were transfer‐printed on a PLGA substrate and encapsulated in a further PLGA layer. Eventually, tungsten electrical connections were patterned between different single transistors as needed. Typical nMOSFET voltage threshold and electron mobility were reported to be about 1.2 V and 630 cm^2^ V^−1^ s^−1^, respectively. Accelerated dissolution experiments on wafer‐attached transistors were performed in PBS at pH 7.4 and 96 °C. Encapsulation layers, interlayer dielectrics, and connection layer dissolved within 5 days, while buried thermal oxide dissolved in the subsequent 10 days. The only insoluble (yet biocompatible) components were adhesion layers (100 nm thick) made of titanium and titanium nitride, whose disintegration happened through delamination, peeling, and flake formation. Device integrity when transferred on PLGA substrate was tested in PBS at pH 7.4 and 70 °C: transistors and interconnections fractured within a few hours, due to PLGA swelling and buckling. PLGA total degradation time was estimated to be around 20 days in the tested conditions (pH 7.4 and 70 °C). Summarizing, bioresorbable silicon‐based transistors have been successfully fabricated with performance comparable to that of standard silicon devices, and the process has been demonstrated to be scaled up to wafer level using industrial IC processes. Although biocompatibility and dissolution of these devices have not always been assessed both in vitro and in vivo (in animal models), the biocompatibility of materials used for transistor fabrication was formerly assessed in independent studies, as discussed in Section 2; performance of isolated devices was only tested in vitro to date.

#### 3.1.2 Organic Transistors

**Figure 4:**
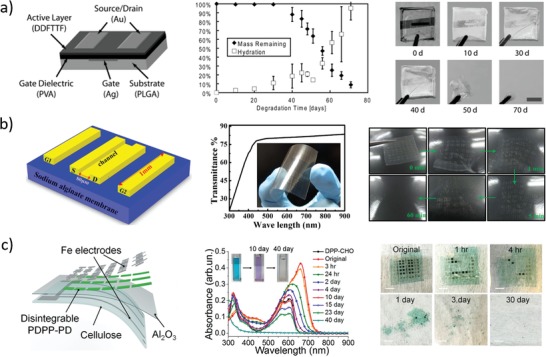
Representative examples of organic transistors. a) Left: Sketch of the organic transistor with biodegradable substrate and gate dielectric.^74^ Middle: In vitro degradation experiments, performed in citrate buffer at pH 4 and 37 °C. Right: Representative photographs of in vitro device degradation; scale bar is 5 mm. Reproduced with permission.^74^ Copyright 2010, John Wiley and Sons. b) Left: Sketch of the biodegradable Electric Double Layer transistor.^79^ Middle: Transmittance spectrum and photograph of a fabricated transistor array, showing transparency and flexibility. Right: Representative photographs of in vitro device degradation in DIW. Reproduced with permission.^79^ Copyright 2015, IEEE. c) Left: Sketch of the ultralightweight and biodegradable synthetic polymer transistor.^80^ Middle: Monitoring of PDPP‐PD degradation in aqueous solution at pH 4.6 by absorbance spectroscopy. Right: Representatives photographs of in vitro device degradation in pH 4.6 solution containing 1 mg mL^−1^ cellulase enzyme. Reproduced with permission.^80^ Copyright 2017, The Authors, Published by National Academy of Sciences.

Following the work of Kim et al.^65^ (2009) on silicon‐based n‐channel MOSFETs on a silk bioresorbable substrate, organic bioresorbable transistors have been also proposed, exploiting well‐known degradable materials commonly used in food, textile, and cosmetic industry (**Figure** **4**).

Bettinger and Bao in 2010 reported on a FET^74^ making use of crosslinked poly(vinyl alcohol) (PVA) as gate dielectric, 5,5'‐bis‐(7‐dodecyl‐9*H*‐fluoren‐2‐yl)‐2,2′‐bithiophene (DDFTTF) as p‐channel semiconductor, and silver and gold for electrical contacts, both of which were not biodegradable, though having good biocompatibility (Figure 4a); PLGA 85:15 was used as substrate. Threshold voltage and mobility of the transistor were about −15 V and 0.2 cm^2^ V^−1^ s^−1^, respectively. Upon immersion in citrate buffer (pH 4.0) at 37 °C, transistor performance immediately started to deteriorate because of DDFTTF delamination from the dielectric, and the device ceased functioning within hours. PLGA structurally started degrading in one month, and the near‐total device resorption occurred approximately in 70 days.

Capelli et al.^75^ proposed silk fibroin as a gate dielectric in n‐type and p‐type FETs, making use of *N,N′*‐ditridecylperylene‐3,4,9,10‐tetracarboxylic diimide (P13) as n‐type semiconductor, α,ω‐dihexyl‐quaterthiophene (DH4T) as p‐type semiconductor, and indium tin oxide (ITO) and gold for electrical contacts; glass was used as substrate. The p‐type FET had a threshold voltage of −17 V and a mobility of 1.3 × 10^−2^ cm^2^ V^−1^ s^−1^, while the n‐type FET had a threshold voltage of 2 V and a mobility of 4 × 10^−2^ cm^2^ V^−1^ s^−1^. For n‐type FET, deep‐red (700 nm) light emission was also achieved at *V*_gs_ = *V*_ds_ = 90 V.

Jeon et al.^76^ proposed chicken albumen as potentially biodegradable gate dielectrics of n‐type FET, further using amorphous In–Ga–Zn–O (IGZO) as semiconductor, alumina as passivation layer, and ITO and aluminum as electrical contacts. Transistors were fabricated using either glass or paper as substrate. The FETs fabricated on glass substrate had threshold voltage and electron mobility of 2.25 V and 5.64 cm^2^ V^−1^ s^−1^, respectively, while the ones on paper substrate exhibited 1.73 V and 6.48 cm^2^ V^−1^ s^−1^, respectively.

Irimia‐Vladu et al.^77^ reported on both p‐ and n‐type semiconductor FETs exploiting glucose, caffeine, or adenine‐guanine bilayers as gate dielectrics (possibly in conjunction with inorganic alumina to increase performance), indanthrene yellow G and indanthrene brilliant orange RF (known in textile and food industry as Vat yellow 1 and Vat orange 3, respectively), β‐carotene, or indigo as semiconductors, and aluminum for electrical contacts. Caramelized glucose and gelatin were used as substrates. Operational voltages and electrical mobilities were in the ranges of 4–20 V and 10^−4^–10^−2^ cm^2^ V^−1^ s^−1^, respectively. Indigo pigment was further investigated by Irimia‐Vladu et al.^78^ in a following work, using anodized aluminum passivated with tetratetracontane (a biodegradable long‐chain alkane commonly found in nature) as gate dielectric, and aluminum for electrical contacts; shellac resin was used as bioresorbable substrate. Indigo showed ambipolar semiconductor properties, enabling the fabrication of both p‐type and n‐type FETs, as well as of complementary MOSFET (CMOS) inverters. The FET threshold voltages were between −1.5 and −3 V for p‐type, and between 4.5 and 7 V for n‐type transistors, while carrier mobilities were around 10^−2^ cm^2^ V^−1^ s^−1^ in both cases. Indigo performance in n‐type FET degraded upon exposure to air, so that encapsulation with polyimide was used to avoid oxygen‐related degradation.

Although the organic materials employed in the fabrication of these transistors were all potentially biodegradable, no degradation, dissolution, or biocompatibility tests were reported for the studies discussed above. Further, the inorganic materials used were not biodegradable, at least under physiological conditions.

In 2015 Guo et al.^79^ proposed an electric double layer transistor with aluminum‐zinc oxide electrodes directly deposited on a sodium alginate membrane acting as both bioresorbable substrate and active layer (Figure 4b). Threshold voltage and carrier mobility were −0.05 V and 6.19 cm^2^ V^−1^ s^−1^, respectively. Upon immersion in DIW at room temperature, sodium alginate rapidly dissolved within 5 min, followed by electrode hydrolysis in 1 h.

In 2017 Lei et al.^80^ reported on pseudo‐CMOS inverters, NAND, and NOR logic ports fabricated using alumina as die‐lectric, a synthetic conjugated polymer as p‐type semiconductor, namely, poly(diketopyrrolopyrrole)‐phenylenediamine (PDPP‐PD), and gold or iron (Fe) for electrical contacts (Figure 4c). Ultrathin regenerated cellulose was used as substrate. The threshold voltage and carrier mobility with gold electrodes were about −4.67 V and 0.21 cm^2^ V^−1^ s^−1^, whereas iron electrodes resulted in slightly poorer performance, namely, about −5.75 V and 0.12 cm^2^ V^−1^ s^−1^, respectively. Upon immersion in acetate buffer at pH 4.6, PDPP‐PD, alumina, and iron spontaneously dissolved; conversely, the use of cellulase enzyme (1 mg mL^−1^) was necessary to degrade the cellulose substrate. The entire device was completely dissolved within 30 days in cellulase‐containing buffer at pH 4.6. PDPP‐PD degradation was further investigated via absorbance spectroscopy in acetic acid solution 1% v/v in water, showing complete depolymerization in 10 days, and total disintegration in 40 days. The biocompatibility of PDPP‐PD was proved through in vitro cell culture with HL‐1 cardiomyocytes on a glass slide coated with the polymer. No significant difference in cell viability was observed after 6 days, compared to cell cultured on a control surface.

Summarizing, biodegradable organic transistors have shown poor electrical performance with respect to bioresorbable inorganic semiconductor FETs, and in most cases device degradation was only postulated and concerned with single elements of the whole devices, such as, semiconductor, substrate, or dielectric layers. Only in few cases degradation has been tested in vitro, and never in vivo, and stability of the performance of such transistors upon immersion in aqueous media has been mostly overlooked.

#### 3.1.3 Passive Components

Bioresorbable passive components (e.g., resistors, inductors, antennas, capacitors, diodes) have also been proposed^28^, ^81^, ^82^, ^83^, ^84^, ^85^, ^86^, ^87^, ^88^ either to complement active components (i.e., transistors) toward the realization of more complex circuits (e.g., antennas in radio frequency (RF) circuits), or to be used as transducers (e.g., heaters) in biomedical applications (e.g., antibacterial therapy).

**Figure 5:**
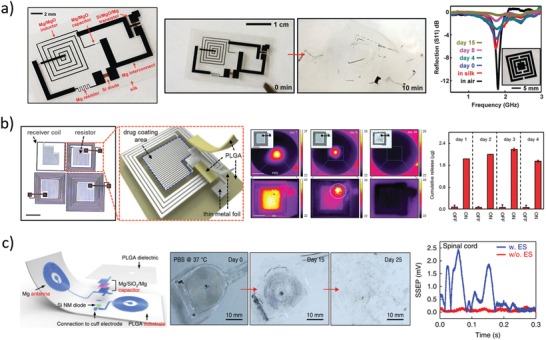
Representative examples of electronic passive components. a) Left: Photograph of various bioresorbable discrete circuital elements on silk substrate.^28^ Middle: Representative photographs of circuit dissolution in deionized water at room temperature. Right: Metamaterial magnesium antenna transmission peak measured during 15 days of implantation in rat. Reproduced with permission.^28^ Copyright 2012, American Association for the Advancement of Science. b) Left: Photograph (scale bar: 5 mm) of four wirelessly actuated heaters and sketch of the device materials and structure.^82^ Middle: Thermographic camera images (inset: optical micrographs) of activated devices during dissolution in PBS at pH 7.4 and 37 °C. Right: Doxorubicin release with and without device activation, showing drug retention in the lipid bilayers for several days if device is not activated. Reproduced under the terms of the CC BY license.^82^ Copyright 2015, Springer Nature. c) Left: Sketch of the double‐coil antenna with half‐wave rectifier circuit for nerve electrical stimulation.^83^ Middle: Representative photographs during device degradation in PBS at pH 7.4 and 37 °C. Right: Somatosensory evoked potentials in rat with and without nerve electrical stimulation via bioresorbable coil and electrodes. Reproduced with permission.^83^ Copyright 2018, Springer Nature.

In 2012, Hwang et al.^28^ reported on the fabrication of inductive coils, resistive serpentines, and capacitive electrodes using Mg for electrical contacts and conductive traces, MgO or SiO_2_ as encapsulation layers, dielectric materials for capacitors and MOSFET gates, and interlayer insulators, Si as active semiconductor in diodes and transistors, and silk as substrate (**Figure** **5**a). Single components were fabricated and tested in vitro, demonstrating functionality and lifetime tunability with encapsulation layer type and thickness: for example, a 300 nm thick Mg serpentine with stable resistance over a time interval tunable from 3 h (400 nm MgO) to 100 h (crystallized silk) in DIW at room temperature was reported. A magnesium coil connected to a silicon resistor was demonstrated as an effective in vitro thermal bactericidal device for an *Escherichia coli* colony in a Petri dish: namely, an external coil was fed with 2 W at 80 MHz for 30 min, causing inductively coupled current flow in the disintegrable circuit and, in turn, resistor heating up to 50 °C. The coil‐resistor circuit was implanted in mouse, resulting in a local temperature increase of about 5 °C upon activation. A metamaterial Mg (400 nm) antenna coated by MgO (600 nm) and encapsulated in silk (100 µm) was implanted in rat and used to monitor biofluid infiltration‐related biodegradation, showing continuous resonance quality factor degradation (Q decreasing from 7 to 2) along 15 days. After this time, the device was retrieved from the animal, showing faint disconnected Mg residues.

In 2014, Tao et al.^81^ used magnesium resistors (200 nm thick) and coils (2 µm thick) on silk substrate (50 µm) to demonstrate thermal effects for direct antibacterial activity and potential on‐demand drug release. The device was characterized in vitro to overheat a bacterial culture on a Petri dish, effectively killing *Staphylococcus aureus* in the heated zone. In vitro degradation of nonencapsulated devices in deionized water resulted in disintegration after 5 min and complete dissolution within 150 min. In vivo implants in rats infected with *S. aureus* resulted in sanitizing the wound with two sets of 10 min activations of the wireless resistor at 49 °C (500 mW input power of the external coil, at 80 MHz). After 7 days from implantation, only few traces of the magnesium coil and silk substrate were visible, which were completely resorbed at day 15. In vitro release of bactericidal drug was also demonstrated by coating the device with an ampicillin‐soaked silk layer, and accelerating its dissolution through resistor‐induced heat.

One year later, Lee et al.^82^ used a very similar approach to fabricate bioresorbable coils for frequency‐addressable drug releasing devices. Molybdenum coils and serpentines were deposited on PLGA substrate, and thermally responsive lipid bilayers were assembled on top, to encapsulate hydrophobic drugs such as antitumoral doxorubicin (Figure 5b). The number of coil turns defined the circuit resonance frequency, while activation time and power (100–1300 mW) modulated the temperature increase to tune lipid bilayer destabilization and, in turn, drug release. In vitro degradation experiments were conducted in PBS at pH 7.4 and 37 °C, resulting in stable operation for 7 days, with significant performance loss occurring on day 8. Complete molybdenum dissolution and PLGA resorption were estimated in several months. Ex vivo experiments with porcine skin showed no doxorubicin leak at 24 h from implantation, and significant drug release after 3 h from device activation at 43 °C. In vivo biocompatibility studies carried out on mice for 5 weeks resulted in immune‐inflammatory response comparable to FDA‐approved HDPE (similar levels of inflammatory cytokines and percentages of immune cells in blood), thus suggesting good biocompatibility of both materials and device.

Very similar coils were also reported by Koo et al.^83^ and Guo et al.^84^ for wireless energy transfer via electromagnetic coupling. In particular, Koo et al.^83^ used a wireless RF receiver to stimulate peripheral nerves in injured rodents (Figure 5c). The device consisted in a magnesium double‐coil antenna (50 µm thick), a silicon RF diode (320 nm silicon with 300 nm Mg electrodes) and a magnesium/silicon oxide/magnesium capacitor (50 µm/600 nm/50 µm), connected so as to form a half‐wave rectifier on a PLGA substrate (30 µm), with magnesium electrodes (50 µm) for contacting the nerves with a wrapping cuff. In vitro dissolution tests in PBS at pH 7.4 and 37 °C showed component disintegration within one month, while in vivo implant residuals were still visible after 8 weeks. The device was tested on living rodents to wirelessly stimulate muscular contraction and accelerate healing of peripheral nerve injury, using the wireless bioresorbable stimulator for 1 h per day, for 6 consecutive days after nerve cut and device implantation.

In 2014, Huang et al.^85^ reported on a transient printed circuit board (PCB) for the assembling of both active and passive components fabricated using rapidly biodegradable materials. The PCB (23 mm × 23 mm) consisted of stacked layers of transient metal traces and dielectric interlayers on a flexible support. Specifically, sodium carboxymethyl cellulose (Na‐CMC, 50 µm thick) was used as substrate, and Mg, W, and/or Zn (2 µm thick) were used for electrical traces. A layer of poly(ethylene oxide) (PEO) (1 µm thick) between the different Na‐CMC layers of the stack bonded them together. A transient metal paste, consisting of either W or Zn microparticles (4 to 12 µm in average diameter) dispersed in PEO in an organic solvent, was used to mount commercial‐off‐the‐shelf (COTS) components onto contact pads located on the PCB. Evaporation of the solvent at room temperature resulted in a solidified metal/polymer composite with good conductivity properties (up to 40 kS m^−1^), which acted as a conductive glue between the pins of the COTS components and the PCB. Dissolution tests of the PCB were carried out in water at room temperature, with polymeric materials completely dissolving in 10 min, and transient metals dissolving on timescales of hours to days. As a proof‐of‐concept application, a RF circuit that sensed the ambient temperature and transmitted the measured data through a frequency‐modulated wireless signal was assembled on a PCB with two stacked layers. A transmission antenna located 30 cm away from the circuit was used as power source. The frequency of the wireless signal transmitted by the circuit increased from 2.47 to 2.49 GHz as the temperature changed from 35 to 18 °C.

In 2015, Hwang et al.^86^ reported on the use of bioresorbable Mg resistors and antennas, and Si NM p–n diodes for the fabrication of bioresorbable passive circuits that changed functionality over time. In a first example, a serpentine Mg resistor was connected in parallel with a Si NM p–n diode, the latter of which was encapsulated with a layer of MgO whereas the Mg resistor was unprotected. At low electrical biases (up to 1 V) most of the current flowed through the resistor, being its resistance much lower than that of the diode. Upon immersion in water, dissolution of Mg occurred, thus changing the electrical response of the circuit from that of a resistor to that of a diode. In a further example, a Mg resistor was connected to a Mg antenna, where specific regions of the antenna were encapsulated with 400 (region 1) and 800 nm (region 2) thick layers of MgO. The other regions of the passive system were encapsulated with bilayers of MgO (800 nm) and SiO_2_ (2 µm). Upon immersion of the system in water, regions 1 and 2 of the antennas progressively dissolved with different times, thus changing length and, in turn, resonance frequency of the antenna from 1.8 GHz (before immersion) to 1.9 GHz (regions 1 dissolved), and eventually to 2.2 GHz (regions 2 dissolved).

In 2017, Lee et al.^87^ reported on a fully biodegradable micro‐supercapacitors (MSCs) with high electrochemical performance. Fe, Mo, or W (about 300 nm thick) were used as electrode materials and current collectors, agarose gel with NaCl salt as hydrogel electrolyte (150 µm), PLGA (about 15 µm thick film) was used as supporting substrate, and polyanhydride as encapsulating material. MSCs with Mo interdigitated electrodes offered an areal capacitance of 1.6 mF cm^−2^ at a current density of 0.15 mA cm^−2^, and areal energy density and power density of 0.14 µW h cm^−2^ and 61 µW cm^−2^, respectively. These values were comparable to those of recently reported nontransient supercapacitors. The electrochemical performance of the supercapacitors was measured up to 10 000 charge/discharge cycles. The capacitance of Mo MSCs increased dramatically during the first 3000 cycles when charged at 0.1 mA cm^−2^, reaching a maximum value of 4 mF cm^−2^ (20 times larger than the initial value), and then decreased. This effect was ascribed to additional redox reactions occurring at the metal oxide growing during the capacitor charge/discharge cycles, until the oxide thickness hindered electronic exchange. The performance of Mo MSCs encapsulated in PLGA (about 15 µm) was also studied upon immersion in PBS 10 × 10^−3^ m at pH 7.4 and 37 °C. The MSCs showed stable performance for 6 h after immersion in PBS, then quickly and completely dissolved afterward (at 10 h). To increase lifetime, an Mo MSC was encapsulated and sealed using polyanhydride (150 µm thick), thus extending stable operation time of the MSC up to 36 h.

### 3.2 Sensors

There is today an increasing demand for biodegradable and bioresorbable sensors to be used in sport&wellness and/or healthcare applications. In this section we will review the main achievements on physical (e.g., pressure, force, temperature) and chemical (e.g., ions, pH, biomolecules) bioresorbable sensors. In the aforementioned milestone work of Roger's group,^28^ silicon strain gauges (gauge factor near 40) and silicon diodes and magnesium thermoresistive sensors (sensitivity of −2.23 mV °C^−1^ and 0.23% °C^−1^, respectively) had been proposed, along with Si photodiodes in a 2D array configuration, opening great possibilities for following developments.

#### 3.2.1 Physical Sensors

**Figure 6:**
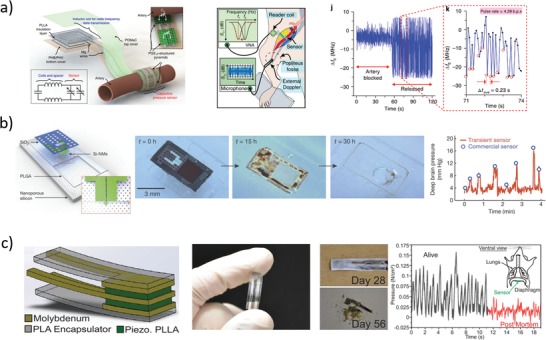
Representative examples of physical sensors. a) Left: Sketch of the structure and functional principle of the wireless‐readable capacitive pressure sensor.^92^ Middle: Sketch of the in vivo inductively coupled measurement setup. Right: In vivo rat heart rate measurement. Reproduced with permission.^92^ Copyright 2019, Springer Nature. b) Left: Sketch of the piezoresistive Si NM pressure sensor.^94^ Middle: Representative photographs of device dissolution during immersion in aqueous buffer at pH 12. Right: Calibration of device performance in artificial cerebrospinal fluid. Reproduced with permission.^94^ Copyright 2016, Springer Nature. c) Left: Sketch of the piezoelectric pressure sensor.^96^ Middle: Photographs of a fabricated device before degradation (left) and after (right) immersion in PBS at 74 °C. Right: In vivo measurement of diaphragm pressure in mouse. Reproduced with permission.^96^ Copyright 2018, The Authors, Published by National Academy of Sciences.

Pressure and temperature sensors represent the most studied bioresorbable physical sensors to date. The former exploit variable capacitors, piezoelectric crystals, and strain gauges; the latter mainly exploit thermoresistive materials (**Figure** **6**).

Luo et al.^89^ fabricated fully bioresorbable capacitors using a PLLA substrate sealed with PCL, with metal connections and electrodes made of iron (adhesion promoter) and zinc. Namely, iron (5–10 µm thick) and zinc (50 µm) were electroplated through a mask in order to fabricate a coil (inductor) connected to two disks (capacitor electrodes); the obtained metal traces were then laminated on a poly(l‐lactide) (PLLA) substrate (200–300 µm), and the assembly was folded so as to obtain a plane capacitor with the metal disks separated by a 30 µm PLLA O‐ring, and sealed with 40 µm PCL spacer. The capacitor was thus enclosed in a passive inductor, capacitor, and resistor (LCR) circuit, and the shift of the resonant frequency of the circuit induced by changes of the capacitance value upon application of a pressure was monitored with an external antenna. The system was characterized in the pressure range 0–20 kPa, showing a sensitivity of about −39 MHz kPa^−1^ (relative capacitance sensitivity 0.05 kPa^−1^) in air. In vitro stability tests were performed on the LCR sensing circuit immersed in aqueous saline solution (NaCl 0.9%) without an external applied pressure, resulting in an increased performance during the first 107 h (sensitivity of −54 MHz kPa^−1^), then followed by rapid performance degradation until hour 170. In vitro degradation of the metal traces was tested using freestanding iron/zinc electroplated electrodes immersed in saline solution at room temperature, resulting in disintegration within 24 h, and almost complete dissolution in roughly 300 h.

Boutry et al.^90^ fabricated a capacitive pressure sensor using iron (2 nm thick, used as adhesion promoter) and magnesium (100 µm) as capacitor electrodes, printed on polyhydroxybutyrate/polyhydroxyvalerate (PHB/PHV, 25 µm) substrates, separated by a PGS (150 µm) dielectric spacer. To improve device recovery time after pressure application and reduce hysteresis, the PGS surface was patterned with micropyramids. The relative capacitive sensitivity was 0.76 kPa^−1^ until 2 kPa, and 0.11 kPa^−1^ from 2 to 10 kPa. A 5 × 4 bidimensional grid of sensors was obtained using orthogonal electrical connections in the top and bottom layers. Blood pulse wave from radial, femoral, and carotid arteries was successfully measured on human volunteers by application of the device on the skin. Material degradation was tested by immersing the device in PBS at pH 7.4 and 37 °C for seven weeks, showing rapid metal dissolution, and rather slow polymeric dissolution, which lasted a few months (after seven weeks, the device retained about 85% of its initial weight). Further, mechanical characteristics of PGS cylinders did not vary significantly during seven weeks of incubation in PBS, due to slow material surface erosion mechanism (instead of rapid bulk erosion, typical in many water‐swelling polymers, like PLGA), suggesting device functional characteristic stability over time.

The same group recently reported a hybrid sensor for simultaneous strain and pressure measurement.^91^ It was composed by a capacitive pressure sensor (similar to that previously described^90^ with Mg electrodes separated by PGS micropyramids) and a capacitive strain sensor (two comb Mg electrodes (100 nm thick) patterned on top of PLLA substrates (50 µm thick) and stacked so that a 50 µm thick PLLA acted as dielectrics). The entire device was encapsulated in poly(octamethylene maleate (anhydride) citrate) (POMaC) and PGS. In‐plane strain caused changes in Mg comb alignment, and, consequently, capacitance variations. The capacitive strain sensor was tested in laboratory environment, showing a sensitivity of about −0.23 pF %^−1^, with a capacitance percentage change of 50% at strain of 15%, with fast response time (within milliseconds), good stability to cycling (capacitance drift about 10% after 20 000 cycles between 5% and 10% strains), and no crosstalk between pressure and strain measurements. The device was tested in vitro by immersion in PBS at pH 7.4 and 37 °C, showing stable performance for 2–3 weeks, with much longer degradation time (after 7 weeks most of the device was still not degraded). In vivo subcutaneous implantation of the sensor in the back of rats resulted in good pressure and strain signal detection after 3.5 weeks, thanks to POMaC and PGS slow surface erosion mechanism. In vivo biocompatibility studies (both immunohistochemistry and H&E staining) carried out over 8 weeks from implantation showed inflammatory response comparable to that of silicone patch used as control.

In another recent work of the same group,^92^ a miniaturized capacitive pressure sensor with slightly different geometry was implanted in rat around the femoral artery, and heart rate was wirelessly measured on‐demand, exploiting RF coupling to monitor LCR resonant circuit frequency shift.^89^, ^93^ Namely, PHB/PHV and POMaC (thickness of 10 µm each) were used as bottom and top substrate, respectively, laser‐cut magnesium foil (50 µm) was used for inductive coil antenna and fringe‐field capacitor metal traces, PLLA (40 µm) was inserted as coil spacer, and micropatterned PGS (40 µm) acted as dielectric (Figure 6a). The sensor was characterized in vitro with both contact and noncontact experiments: for contact measurements, a PDMS slab was pressed (up to 20 kPa) on the device, while in noncontact measurements the PDMS slab was only approached to the device without touching it. In vitro sensor characterization was carried out using an LCR meter directly wired to the device, resulting in a relative sensor sensitivity of about 2.2% kPa^−1^ for contact pressure experiments (in the 0–6 kPa range) and 2.5% nm^−1^ for noncontact experiments. Noncontact mode was of chief importance because a tight wrapping of the device around blood vessel could hinder blood flow inside it. The device was implanted in rat, wrapping femoral artery with softer POMaC layer facing blood vessel wall, and harder PHB/PHV facing outside, in order to reduce pressure interferences due to respiration movements. Rat heart rate was successfully measured by inductive coupling between the internal coil and an external antenna connected to a vector network analyzer, monitoring real‐time resonance frequency shifts. One week after implantation, the signal had a poorer quality, but it was still recognizable. Partial bioresorption (only PHB/PHV left) was observed after 12 weeks without severe inflammation signs, through immunohistochemistry analysis with CD68, a surface marker of macrophages.

Besides variable capacitors, piezoresistive materials that experience resistance changes when subjected to mechanical strain (piezoresistive effect) have been used for bioresorbable pressure sensors. As piezoresistive materials are also (often) sensitive to temperature changes, the same piezoresistive serpentine has been used as temperature sensor if deposited on a rigid surface, and as strain sensor if constrained on a flexible membrane.

In 2016, Kang et al.^94^ fabricated silicon serpentines (for both pressure and temperature monitoring) by patterning Si NMs (300 nm thick) on a SOI wafer and subsequent transfer‐printing on a flexible PLGA membrane (30 µm thick). For pressure sensing, the latter was suspended over a square cavity (about 30–40 µm deep) etched into a nanoporous silicon layer (about 60–80 µm thick, 71% porosity) used as supporting substrate (Figure 6b). Magnesium and molybdenum were used for metal traces, and SiO_2_ was used for electrical insulation and as encapsulating material. Silicon serpentine fabricated outside the cavity area served as temperature sensor. The pressure sensor was calibrated in vitro using artificial cerebrospinal fluid (ACSF), resulting in strain gauge resistance around 258 kΩ, and sensitivity around 0.6 kΩ kPa^−1^, corresponding to a gauge factor of 30. Similarly, temperature sensor in ACSF showed a sensitivity around 0.1 kΩ °C^−1^, with a resistance of 74 kΩ at 35 °C, corresponding to a thermal coefficient about 0.14% °C^−1^. Device dissolution studies were performed in vitro in buffer solution at pH 12 and room temperature, leading to complete dissolution in about 30 h. To increase the device functional time, a polyanhydride encapsulation was also used, at the cost of a decrease in pressure sensor sensitivity (0.38 kΩ kPa^−1^). The bioresorbable pressure and temperature sensors were implanted in living rat brain and wired to an external non‐bioresorbable power supply and communication unit fixed on the scalp, successfully proving intracranial pressure and temperature measurement for 3 and 6 days, respectively, and showing no adverse effects in the animal. Biocompatibility studies of polyanhydride were performed 2, 4, and 8 weeks after implantation through H&E staining, showing no overt adverse immune response from rat brain cells, compared to HDPE used as control. An alternative to the external wiring was proposed by using a commercial non‐biodegradable NFC chip subcutaneously fixed on a PLGA substrate with magnesium electrical connection.

Besides pressure and temperature, also acceleration, thermal conductivity, and flow rate sensors were fabricated by Kang et al.^94^ using serpentine sensors based on patterned Si NMs. Full dissolution, characterization, and calibration of these sensors were not detailed, though their output signals were reported to be comparable to those of commercial non‐bioresorbable control sensors. For instance, an acceleration sensor was fabricated with a piezoresistive strain gauge transfer‐printed on a PLGA cantilever suspended on a cavity etched in the porous silicon substrate. Deflection of the PLGA cantilever, upon an acceleration solicitation, resulted in change of the strain gauge resistance, that was monitored as output signal. Further, a thermal conductivity sensor was achieved using a silicon serpentine resistor. Upon injection of a current through the silicon resistor, its temperature rapidly increased; on the other hand, when the current flow ceased, the temperature of the silicon resistor returned to its initial value with a dynamic that depended on the thermal conductivity of the surrounding medium. Proof‐of‐concept demonstration was given in water, ethylene glycol, toluene, and hexane, though no calibration was reported. Eventually, a flow rate sensor was achieved using two temperature sensors with a silicon resistor in between, spatially arranged in a linear fashion. When the resistor was heated through injection of a current, a temperature gradient arose between the two sensors aligned with the flow direction of the liquid, so that the difference between the temperature sensors was directly related to the fluid flow rate. The sensor was tested in a water bath up to 3 mL s^−1^ showing a sensitivity around 0.15 °C s mL^−1^.

Shin et al.^95^ reported on a piezoresistive pressure sensor fabricated using silicon strain gauges on a suspended amorphous silica membrane. Two SOI wafers with a 100 nm thick top silicon layer (Si NM) were bonded together using a 3 µm thick PDMS adhesion interlayer. Before bonding, four silicon meander‐shaped resistors (defining the pressure and temperature gauge sensors) were defined on the Si NM of one the SOI wafers, and a square trench with size 200 × 200 × 10 µm (defining the pressure sensitive region) was etched on the Si NM of the other one. Two of the silicon resistors, the ones designed to work as pressure‐sensitive strain gauges, were placed in correspondence of the pressure sensitive region; the other ones, designed to work as temperature‐sensitive gauges, were placed on rigid region of the wafer. Thermal treatment at 550 °C for 2 h calcined the PDMS adhesion layer yielding an amorphous silica layer (about 200 nm thick), which acted as a flexible membrane in the pressure sensitive region, where the silica layer was suspended on the square trench. Chemical removal of the back‐side silicon of SOI wafers resulted in ultrathin, bioresorbable electronic devices encapsulated in thermal SiO_2_ layers. The two pressure‐sensitive strain gauges (on the suspended silica membrane) were connected with the two silicon pressure‐sensitive gauges in a Wheatstone bridge configuration, so as to obtain a bioresorbable pressure sensor chip with automatic rejection of thermal effects on pressure measurements. The pressure sensor chip was calibrated in ACSF at pH 7.4 and 37 °C, showing a pressure sensitivity around 0.13 Ω mmHg^−1^ (corresponding to 0.98 Ω kPa^−1^), and good signal stability (variation in pressure sensitivity within 1.5%) after 22 days of continuous operation. Temperature gauges were calibrated in a similar way in ACSF, resulting in a temperature coefficient of resistance (relative sensitivity) of 0.12% °C^−1^. Accelerated dissolution tests were performed in vitro in PBS at pH 7.4 and 95 °C, showing that the pressure sensor chip was completely dissolved within 80 h, with only the thermal SiO_2_ layer of the bottom SOI wafer left after this time; dissolution time in physiological conditions was estimated to be about 400 days. The pressure/temperature sensor chip was implanted in living rat skull and connected to an external digital multimeter to record voltage data. Commercial clinical pressure and temperature sensors were also implanted and used as control. In vivo pressure (while compressing and releasing the rat flank) and temperature (while applying heating blanket or ice pack to the rat) measurements recorded over a period of 25 days were consistent with clinical control sensors, though sensor accuracy degraded with time, and signals disappeared after day 25 (possibly due to dissolution of metal Mo electrodes). The authors further demonstrated wireless pressure data transmission to a personal computer (PC) from a miniaturized potentiostat wired to the implanted sensor chip and externally secured on rat skull. Insights into the physiological reactions to the proposed pressure/temperature sensor chip were achieved through implantation in rats of a miniaturized version (750 µm × 750 µm × 11 µm vs 1.3 mm × 1.3 mm × 16 µm) of the chip without thermal SiO_2_ encapsulation. Evaluation of the biodistribution of dissolved silicon resulted in silicon accumulation mostly in kidney, liver, and spleen after one week, and normal level recovery within 5 weeks; no adverse effects were observed in blood analysis and in histological analysis of brain, spleen, heart, and kidney tissues, by comparison between mice with and without implanted sensor.

In 2018, Curry et al.^96^ fabricated a bioresorbable piezoelectric pressure sensor using PLLA as piezoelectric material. The sensor structure (5 mm × 5 mm in size, 200 µm thick) consisted of two layers of piezoelectric PLLA (27 µm) sandwiched between Mo or Mg electrodes and encapsulated in normal PLA (Figure 6c). To make PLLA piezoelectric, a PLLA film was stretched at an annealing temperature of 90 °C, so as to simultaneously induce polymer chain orientation and improve material crystallinity. The PLLA film was eventually cut with an angle of 45° with respect to the stretching direction to maximize piezoelectric response. PLLA films with different draw ratios (between final stretched length and initial length) were investigated to assess piezoelectric response (through both impact and vibration testing), showing optimal piezoelectrical response of PLLA sensors with a draw ratio around 5 (for which crystallinity is about 55%). The PLLA pressure sensor was then connected to a charge amplifier circuit to convert force‐induced charge to voltage, and calibrated with predefined weights in the range 0–18 kPa. The calibration curve showed two linear regions, namely, 0–2 kPa with sensitivity of 75 mV kPa^−1^ and 3–18 kPa with sensitivity of 14 mV kPa^−1^. Performance degradation of the PLLA pressure sensor was studied both in vitro and in vivo by placing the device in PBS at pH 7.4 and 37 °C and subcutaneously implanting the device in the back of mice, respectively. In both cases, sensor output signals were comparable before and after 4 days of tests, though after 8 days from implantation there was no detectable signal. Accelerated degradation tests were then performed in PBS at pH 7.4 and 74 °C, still showing visible device fragments after 56 days. In vivo subcutaneous implantation in the back of mice showed mild immune response after 2 weeks, which reduced to normal levels after 4 weeks. Histological staining with H&E was performed to observe inflammatory cell response, while Masson's Trichrome blue staining was carried out to detect fibrosis; immunohistochemical staining with CD64 antibody was performed to reveal macrophages. As a proof‐of‐concept application, the PLLA pressure sensor was fixed to mouse abdomen and used to measure diaphragmatic contraction of the living animal during anesthesia and subsequent euthanasia via anesthetic overdose.

In 2017, Salvatore et al.^97^ reported on a biodegradable thermoresistive sensor fabricated by using a magnesium serpentine resistor encapsulated in a compostable, water‐resistant biopolymer, namely, Ecoflex. The magnesium serpentine (10 µm width, 250 nm thick), which was patterned in a fractal design that ensured stretchability of the sensor structure, was sandwiched between silicon dioxide (100 nm) and silicon nitride (100 nm) layers that provided electrical insulation, and eventually encapsulated between two thin films (about 16 µm in total) of Ecoflex. The sensor was calibrated on a hotplate, exhibiting a linear behavior in the temperature range 20–50 °C without significant hysteresis and with absolute sensitivity of about 70 Ω K^−1^ (relative sensitivity about 0.2% K^−1^). Sensor resistance was almost insensitive to folding, crumpling, and stretching (variations less than 10%), envisaging wearable and implantable applications. The sensors was tested in vitro in 150 × 10^−3^ m saline solution at 25 °C, resulting in reliable continuous temperature measurement for 1 day with a resistance variation of 0.6%; electrical connectivity was lost after 28 days, and complete dissolution of the SiO_2_/Mg/SiN_3_ stack occurred in 67 days. The above‐described design was extended to the fabrication of an array of sensors to demonstrate scalability of the approach and broadening of applications. An array of temperature sensors (9 in total) was then integrated onto an Ecoflex fluidic device (overall size 2.8 × 2.5 cm^2^) to perform a 2D mapping of the temperature distribution. Injection of a warm liquid (35 °C) into the channels and simultaneous monitoring of the sensor array response proved that temperature distribution and flow directions matched those measured with a handheld infrared camera. The possibility of wireless monitoring was also demonstrated using a standard non‐biodegradable microcontroller unit with Bluetooth connectivity, wired to the sensor with Ecoflex‐encapsulated zinc wires.

#### 3.2.2 Chemical Sensors

Although the concept of chemical sensors operating in vivo is very appealing, bioresorbable chemical sensors have been limited so far to the detection of ions or small organic molecules, probably because of the challenge of enabling reliable and specific sensing of larger and more complex (bio)molecules in vivo.

**Figure 7:**
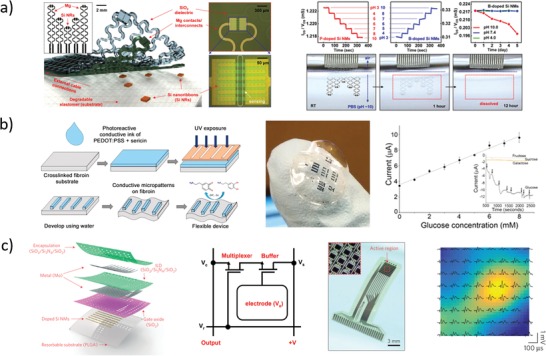
Representative examples of chemical sensors and biopotential electrodes. a) Left: Sketch and photographs of the conductometric pH sensor.^70^ Right Top: Calibration of p‐doped (left) and n‐doped (middle) Si NR sensors, and sensor stability when immersed in aqueous solutions at different pH values (right). Right Bottom: Representative photographs of device dissolution during immersion in PBS at pH 10. Reproduced with permission.^70^ Copyright 2015, American Chemical Society. b) Left: Sketch of the fabrication process of the silk‐based electrochemical electrode.^98^ Middle: Photograph of a fabricated sensor on flexible silk substrate. Right: Calibration of the glucose biosensor in ferrocene‐containing solution. Reproduced with permission.^98^ Copyright 2016, Elsevier. c) Left: Sketch of the actively multiplexed transistor array.^101^ Middle‐Left: Electrical scheme of a single sensing electrode cell. Middle‐Right: Photograph of an 8 × 8 array of multiplexed electrodes. Right: Spatiotemporal mapping of somatosensory evoked potentials in anesthetized rat. Reproduced with permission.^101^ Copyright 2016, Springer Nature.

In 2015, Hwang et al.^70^ proposed a bioresorbable pH sensor by using FET‐like structures based on silicon nanoribbons (Si NRs). Si NRs (length 500 µm, width 40 µm, thickness 300 nm) with p‐type and n‐type doping (about 10^20^ cm^−3^) were defined on a SOI wafer, provided with Mg (300 nm) source and drain electrodes (and electrical interconnections), and coated with SiO_2_ (300 nm) acting as interlayer dielectric in the Si NR sensing regions. A further thin layer of SiO_2_ encapsulated the devices, except for Si NR gate and electrode connection pads. The FET‐like Si NRs were then transfer‐printed on a POC flexible substrate (**Figure** **7**a). The SiO_2_ surface of the nanoribbons was functionalized with 3‐aminopropyltriethoxysilane (APTES), which acted as pH‐sensitive probe. In fact, at low pH values, the −NH_2_ (amine) groups of the APTES underwent protonation (−NH_3_^+^), while at high pH values the −SiOH (silanol) groups of the silicon dioxide surface underwent deprotonation (−SiO^−^). The resulting changes in the electrostatic charge of the SiO_2_ layer with pH modulated the conductance of the Si NRs by depleting or gathering charge carriers: in p‐doped Si NRs (boron, 10^20^ cm^−3^) silicon conductance increased with pH with a sensitivity of 0.3 µS pH^−1^, whereas in n‐doped Si NRs (phosphorus, 10^20^ cm^−3^) silicon conductance decreased with pH with a sensitivity of −0.1 µS pH^−1^, when immersed in PBS 0.1 m at pH ranging from 4 to 10. Operation lifetime of these devices for pH measurements was investigated with boron‐doped Si NRs during immersion in PBS 0.1 m at pH from 4 to 10 over 5 days at 37 °C. At pH 7.4 the conductance of the Si NRs changed by less than 1% during immersion for 5 days. Conversely, unencapsulated Si NR pH sensors exhibited similar changes (less than 1%) after just one day in such conditions. At higher pH (i.e., 10), a linear decrease in conductance (less than 10%) during this period occurred, as expected. Degradation experiments of unencapsulated Si NR pH sensors were performed by immersion in PBS at pH 10 at room temperature: in 12 h magnesium traces disappeared completely, while POC, Si, and SiO_2_ slowly dissolved in several weeks. The same approach was used by the same group to fabricate a pH sensor by functionalizing phosphorus‐doped silicon nanoribbons on a PLGA substrate, obtaining a sensitivity around −0.3 µS pH^−1^.^94^

An organic, bioresorbable (yet not completely) electrochemical biosensor for specific detection of glucose was reported by Pal et al.^98^ (Figure 7b). A poly(3,4‐ethylenedioxythiophene)‐poly(styrene sulfonate) (PEDOT:PSS) copolymer (conductive material) was dispersed (19%) in silk sericin‐based photoresist (SPP) to obtain a conductive photocurable ink. By adding 200 U of glucose oxidase (GOx) enzyme to the SPP‐PEDOT:PSS conductive ink, glucose‐selective electrodes were patterned on flexible and biodegradable fibroin protein photoresist (FPP) films. The analytical performance of the electrochemical biosensor was tested in PBS 100 × 10^−3^ m at pH 7.4, with ferrocenyl methyl trimethyl ammonium iodide (0.1 × 10^−3^ m in solution) as redox mediator, at different glucose concentrations. The calibration curve measured for glucose concentrations in the range 0–8 × 10^−3^ m showed a linear behavior with sensitivity of 7.57 µA mm ^−1^ cm^−2^ and limit of detection of 1.16 × 10^−3^ m. Good selectivity was achieved against other sugars, namely, fructose, galactose, and sucrose (100 × 10^−3^ m in solution). GOx‐functionalized sensors were claimed to be stable over time and to operate for 2 weeks after preparation. Degradation experiments on bare SPP‐PEDOT:PSS electrodes (i.e., without GOx enzyme) on FPP were performed in PBS 10 × 10^−3^ m containing 10 U of protease enzyme, resulting in SPP and FPP complete dissolution in 4 weeks. The PEDOT:PSS copolymer was not biodegradable (though biocompatible), and remained as fibrous strands in the solution.

Recently, Kim et al.^99^ proposed a flexible, bioresorbable electrochemical sensor that employed Si NMs coated with iron‐containing nanoparticles as catalyst to enable the detection of neurotransmitters (i.e., dopamine (DA)). The sensor consisted of highly doped (boron, 10^20^ cm^−3^) crystalline Si NMs (300 nm thick) uniformly decorated with hybrid nanoparticles (NPs) of iron (5 nm) and carboxylated polypyrrole (CPPy, 100 nm) as active sensing elements. Chromium/magnesium metal traces (10/200 nm thick) were employed for electrical contacts and interconnections, and a layer of SiO_2_ (100 nm thick) isolated the conducting Mg traces from biofluids and adjacent tissues, apart from the sensing area. As a supporting element, a thin and flexible sheet of PCL was used. Investigation of the detection mechanism highlighted that: dopamine molecules were adsorbed onto the CPPy surface of the hybrid NPs (Fe^3+^_CPPy NPs) via π–π interactions; Fe‐based NPs catalyzed oxidation of DA accumulated on CPPy to form DA‐derived quinone; eventually, electrons generated from the oxidation reaction were transferred to highly p‐doped Si NM‐based electrodes producing a change in their electrical characteristics. The sensor was tested in vitro in PBS 10 × 10^−3^ m, showing good sensitivity toward dopamine (a concentration as low as 1 × 10^−12^ m was clearly detected), with high selectivity against interferents and other neurotransmitters, e.g., ascorbic acid, uric acid, epinephrine and norepinephrine (no appreciable responses were recorded at concentration of 1 × 10^−3^ m each). Selectivity was explained in terms of π–π interactions between polypyrrole and DA, not likely to happen for the other molecules tested. The electrochemical device spontaneously and fully dissolved in 15 h when immersed in PBS 10 × 10^−3^ m at pH 11 and 37 °C (equivalent to 5 days at pH 7).

An open challenge on bioresorbable chemical sensors and biosensors, with respect to physical sensors, is related to the sensor protection from dissolution during operation lifetime. In fact, differently from physical sensors, in chemical sensors the sensing material is required to have direct contact with the fluid of interest containing the target analytes, so that they cannot be fully encapsulated with a barrier material. The research on new bioresorbable polymers to be used as sensing material in chemical sensors with optimal characteristics in terms of tunable dissolution is still ongoing.^51^

#### 3.2.3 Electrodes for Biopotentials

Bioresorbable materials, either metallic or semiconducting, have been also used as biopotential sensors with the aim of revealing abnormalities in bioelectric signals related to severe neurological diseases.

Campana et al.^100^ demonstrated a biocompatible, organic electrochemical transistor able to measure electrocardiographic signals. PLGA (20 µm thick, 75:15 lactide:glycolide) was used as a flexible substrate, on top of which drain and source gold electrodes (30 nm thick, length 30 µm, and width 1000 µm) were patterned. PEDOT:PSS (200 nm thick) was patterned on top of electrodes and employed as organic active material of the transistor, for which biopotential was used as gate signal by directly contacting skin surface with the polymer. Characterization of the electrochemical transistor was carried out (also in the presence of mechanical stress, i.e., bending the transistor down to a curvature radius of 80 µm) by casting in the channel region a drop of PBS 0.1 m at pH 7 and room temperature, in which a Pt or Ag wire was immersed and used as the gate electrode. Output curves and transfer characteristic agreed with that expected for a thin film transistor, with low sensitivity to mechanical stress. Assessment of the potentiometric recording capabilities of the bioresorbable electrochemical transistor in a medically relevant setting was achieved by recording a human electrocardiogram (ECG) by attaching the exposed PEDOT:PSS transistor channel directly to skin, using commercial electroconductive gel to promote adhesion and to reduce impedance with skin. In this configuration, the skin replaced the metal gate electrode, so that skin potential changes with respect to a ground contact led to transient fluctuations of the drain current. When operated at *V*_SG_ = 0.5 V, with respect to the grounded body, and *V*_SD_ = −0.3 V (in order to work with high transconductance and low leakage current), the recorded current trace contained the typical spikes of the heart beat with an amplitude of ≈100 nA, which agreed well with spikes of 500 µV measured with traditional potentiometric recording. The results highlighted the feasibility of realizing simple organic bioelectric interfaces on implantable bioscaffolds, which would allow the recording of signals from muscular or nervous tissue to monitor health state, or which could provide electrical stimulation to influence the tissue activity.

Hwang et al.^70^ used stretchable Mg (300 nm) serpentines, with SiO_2_ as interlayer dielectric and encapsulation layer, transfer‐printed on POC flexible substrate, as capacitive electrodes for precordial ECG and forearm electromyogram (EMG) recording in human volunteers. The Mg electrodes were coupled to the skin through the thin sheet of POC, and the sensing mechanism relied on displacement currents generated by capacitive coupling through the SiO_2_ interlayer. The resulting ECG and EMG data were quantitatively comparable to those obtained with conventional gel electrodes. Degradation tests performed in PBS at pH 10 and room temperature showed that Mg and SiO_2_ completely dissolved within hours (Mg) or days/weeks (SiO_2_).

One year later, Yu et al.^101^ reported on the use of bioresorbable Si NMs with high doping levels for the fabrication of multiplexed passive and active electrodes for neural signal recording. An array of thin, flexible, passive electrodes was fabricated by patterning phosphorus‐doped (10^20^ cm^−3^) Si NMs (thickness 300 nm), which were then transfer‐printed on a flexible sheet of PLGA (thickness 30 µm) used as substrate. A layer of SiO_2_ (thickness 100 nm) coated the Si connection traces to isolate them from biofluids and adjacent tissue, except for the terminal pads where Si was exposed and used as direct neural interface. Bioresorbable neural electrode arrays with several measurement channels, namely, 4 and 256 (in 16 × 16 configuration, overall area 3 cm × 3.5 cm), were reported. In vitro characterization of passive electrodes was carried out by immersing the electrodes (placed on a hydrogel substrate) in PBS at pH 7.4 and room temperature, and monitoring the electrode impedance versus operation frequency (through impedance spectroscopy measurements, with a gold electrode as standard reference). Experimental results showed that the performance of Si NM electrodes was comparable with that of the Au electrode. In vitro degradation tests highlighted that complete dissolution of the passive electrodes was achieved in 4–5 weeks in PBS at pH 7.4 and 37 °C. Passive electrodes were used for in vivo neural recording experiments in anesthetized adult rat. A craniotomy exposed a 4 × 8 mm^2^ region of cortex in the left hemisphere and the Si NM electrodes (four channels) were positioned on the cortical surface for recording physiological oscillations under isoflurane anesthesia, next to a standard stainless‐steel microelectrode used as a control. Experimental results showed that epileptiform spiking activity induced by application of crystals of bicuculline methoxide was successfully recorded by the bioresorbable electrodes, in agreement with the control electrode, also with high signal‐to‐noise ratio (42 for bioresorbable, 32 for control electrode). The bioresorbable electrodes were also implanted on the periosteum and used for high‐fidelity recording of electroencephalogram (EEG) and evoked potentials, successfully capturing theta waves (highlighted in power spectral analysis of the recorded traces) and sleep spindles. Further chronic tests of electrocorticogram (ECoG) recording indicated long‐term stability of operation for more than one month without significant performance change, using electrodes with increased thicknesses of SiO_2_ (300 nm) and Si NMs (1000 nm). Sudden failure occurred at day 33, probably due to an open circuit state of interconnections. Studies of tissue reactions to bioresorbable electrode arrays involved chronic implants and were carried out in 14 animals, using Pt electrodes with similar geometries as control. Immunohistochemical analyses showed no significant tissue inflammation at the implantation site, for both Si NM and control electrodes, when compared to the control contralateral hemisphere.

Similar passive electrodes were fabricated by and Lee et al.^33^ using Mo traces for electrical connection, instead of a single long Si nanomembrane; SiO_2_ was used as encapsulation layer, leaving openings for Si recording regions.

Yu et al.^101^ also reported on arrays (8 × 8 configuration) of active electrodes fabricated by using a similar technology (Figure 7c). Namely, 128 bioresorbable transistors were fabricated using Si NMs transfer‐printed on a PLGA sheet, employing SiO_2_ (100 nm thick) as gate dielectric, Mo traces (thickness 300 nm each) for electrical connections and sensing gate electrodes, and a stack of SiO_2_/Si_3_N_4_/SiO_2_ (thickness 300/400/300 nm) as interlayer dielectric and encapsulation layer, with openings for Mo sensing gate electrodes. Every electrode cell was composed of two nMOSFETs, namely, a buffer transistor and a selection transistor, in order to obtain a densely packed electrode array with reduced external connections enabling multiplexed reading. In vitro accelerated degradation of the multiplexed electrode array was investigated in PBS at pH 12 and 37 °C, and allowed to estimate a degradation time in physiological conditions (pH 7.4) of about 4–6 weeks for PLGA, Si, and Mo, and 6 months for SiO_2_ and Si_3_N_4_. Eventually, the multiplexed electrode array was used in vivo to measure 2D ECoG in anesthetized rats. Drug‐induced epileptic spikes were successfully recorded, allowing spatiotemporal mapping of different neural wave propagation, highlighting spiral, diagonal, and lateral sweeps. In addition to pathological condition, physiological somatosensory evoked potentials were also recorded on barrel cortex under whisker stimulation, giving amplitude spatial distributions consistent with the activation zone of the solicited whisker. No in vivo degradation tests were reported for multiplexed active electrode arrays.

### 3.3 Power Supply

**Figure 8:**
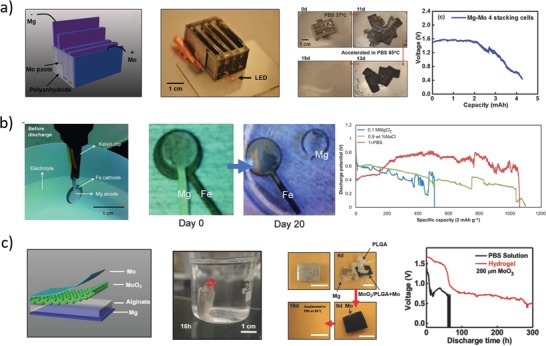
Representative examples of batteries. a) Left: Sketch of the structure of the Mg–Mo battery.^103^ Middle‐left: Photograph of the 4 cell Mg–Mo battery used for powering a LED. Middle‐right: Photographs of the battery degradation during immersion in PBS at 37 °C and 85 °C. Right: Battery discharge under a constant current density of 100 µA cm^−2^. Reproduced with permission.^103^ Copyright 2014, John Wiley and Sons. b) Left: Photograph of the PCL‐encapsulated liquid‐harvesting battery.^104^ Middle: Representative photographs of battery degradation during immersion in PBS 1× at 37 °C. Right: Battery discharge in different aqueous solutions under a constant current density of 230 µA cm^−2^. Reproduced under the terms of the CC BY license.^104^ Copyright 2015, Springer Nature. c) Left: Sketch of the structure of the layered‐cathode battery.^108^ Middle‐left: Photograph of continuous powering of a LED after 16 h of immersion in PBS. Middle‐right: Representative photographs of battery degradation during immersion in PBS at 37 °C and 85 °C. Right: Battery discharge under a constant current density of 25 µA cm^−2^. Reproduced with permission.^108^ Copyright 2018, John Wiley and Sons.

Nowadays, an increasing interest toward organic, harmlessly degradable, and even ingestible batteries is rising.^102^ Bioresorbable power sources, either batteries or energy harvesters, represent indeed an essential component of implanted bioresorbable devices, regardless of the targeted applications (**Figure** **8**).

#### 3.3.1 Batteries

In 2014, Yin et al.^103^ developed a biodegradable battery making use of biodegradable metals for electrodes, and filled with PBS as electrolyte (Figure 8a). Mg was chosen as anode material because of its high energy density and long shelf‐life (when not immersed in water), while Fe, W, or Mo were used as cathode materials, thus achieving Mg‐X single cell batteries (X = Fe, W, or Mo). The operating voltages were about 0.75, 0.65, and 0.45 V for Fe, W, and Mo, respectively, at discharging current of 0.1 mA cm^−2^, and were stable for at least 24 h. A 1 cm^2^ Mg–Mo single cell battery containing 8.7 mg of magnesium (50 µm thick) and 8.2 mg of molybdenum (8 µm thick) showed an energy capacity of about 2.4 mA h, when discharged at 0.1 mA cm^−2^ for 24 h. Due to the corrosion of Mg foils during operation, the measured capacity was significantly lower than the theoretical capacity of Mg (2.2 A h g^−1^). In fact, at the magnesium anode two oxidation reactions occurred simultaneously, namely, current‐driven oxidation (Mg→ Mg^2+^ +2e^−^) and spontaneous water‐driven oxidation (Mg + 2H_2_O→ Mg(OH)_2_ + H_2_). Spontaneous dissolution of magnesium in water reduced lifetime of the battery when filled with PBS, compared to that of nonactivated battery (i.e., Mg in air), leading to rough halving of the battery energy capacity in 1–2 days after PBS filling. Four Mg–Mo cells were stacked together and encapsulated in a polyanhydride layer, in order to achieve a stable (up to 6 h) output voltage of about 1.6 V, at a discharging current of 0.1 mA cm^−2^. As proof‐of‐concept applications, the stacked battery was used to power a RF circuit and a conventional LED. Battery dissolution experiments carried out by immersion in PBS at 37 °C showed that the polyanhydride degraded after 11 days, leaving partially dissolved Mg and Mo foils; accelerating the dissolution by increasing the temperature to 85 °C resulted in a complete battery dissolution after further 8 days.

One year later, Tsang et al.^104^ proposed a bioresorbable battery based on the employment of electrolytes usually available in the human body, namely, MgCl_2_, NaCl, and PBS, in combination with PCL polymer as separator (Figure 8b). Magnesium was used as anode and iron as cathode, and the electrodes were separated by and encapsulated in PCL (5 µm thick). Battery performance in various body fluids was investigated by immersion in MgCl_2_ (0.1 m), NaCl (0.9% by weight), and PBS solutions (1× at pH 7.4) at a discharging current density of 230 µA cm^−2^. Output voltages around 0.5, 0.4, and 0.7 V, and lifetimes of 49, 90, and 99 h, respectively, were achieved, corresponding to specific capacities of 509, 1100, and 1060 mA h g^−1^. Dissolution experiments were carried out in PBS at 37 °C, showing almost complete magnesium dissolution and device delamination after 20 days, while iron electrode dissolved at a much slower rate, and could last from months to years.

To tackle relatively short lifetimes and low power densities of batteries using liquid electrolytes and operating in physiological conditions, Jia et al.^105^ proposed a battery that exploited biocompatible (yet not totally bioresorbable) ionic liquid polymer electrolytes. A magnesium‐aluminum‐zinc alloy (AZ31, 200 µm thick) was used as anode material, poly(pyrrole)‐*para*(toluene sulfonic acid) (PPy‐*p*TS, 40 µm tick) was used as cathode material, and chitosan‐choline nitrate (CS−[Ch][NO_3_]) was used as polymer electrolyte. Eventually, a thin layer of CS−[Ch][NO_3_] solution was dropped onto the assembled battery to hold the device components together (like a glue) and improve mechanical integrity. A 1 cm^2^ (60 µm thick) single cell battery exhibited a high output voltage (1.33 V) and long stability (160 h) when discharged at 10 µA cm^−2^, though performance rapidly degraded at higher discharge currents due to the low ion mobilities, and no stable operation was achieved above 1 V at 0.1 mA cm^−2^. No dissolution tests were performed on this battery. The same group next developed biodegradable batteries that used an AZ31 foil anode and either PPy‐*p*TS^106^ or gold nanoparticles^107^ deposited on a silk fibroin substrate as cathode. In the latter work, silk fibroin−choline nitrate (SF‐[Ch][NO_3_]) was used as polymer electrolyte.^107^ When discharged at 5 µA cm^−2^, a 1 cm^2^ single cell battery exhibited a capacity of 2.2 mA h cm^−2^ with a plateau voltage of around 1 V. To optimize the battery for implantation,^107^ AZ31 alloy was sputtered on crystallized silk fibroin (instead of using a rigid AZ31 alloy foil) and further encapsulated in crystallized silk by thermal processing. The encapsulated battery showed a specific capacity of 0.06 mA h cm^−2^, when discharged at 10 µA cm^−2^, which was lower than that of the unsealed battery (1.43 mA h cm^−2^), due to both rapid depletion of AZ31 and limited oxygen availability. A stable open‐circuit voltage (OCV) of 1.21 V for about 180 min was achieved for the encapsulated battery in air, likely due to the corrosion of the AZ31 layer at the interface with the polymer electrolyte. When exposed to PBS, a two‐stage transient behavior was observed, with a stable OCV above 1.21 V for 64 min (with same discharge profile as in air), followed by a rapid functional degradation in 22 min, explained by a failure in the crystallized silk protection film due to swelling upon water adsorption. The overall encapsulated battery (170 µm thickness) almost totally degraded after 45 days, with AZ31 thin film (500 nm thickness) completely disappearing in 4 h. The SF‐Au film 0.09 mg Au in 230 mg total device) was physically fragmented in PBS solution because of the degradation of the silk substrate, and Au nanoparticles (being not biodegradable, though considered nontoxic) were possibly cleared by renal excretion in a potential implantation in living humans.

More recently (2018), Huang et al.^108^ developed a magnesium‐molybdenum oxide (Mg–MoO_3_) bioresorbable battery with extended lifetime using calcium crosslinked alginate in PBS as electrolyte‐retaining polymer (Figure 8c). The anode was a magnesium foil (200 µm) and the cathode consisted of MoO_3_ nanoparticles mixed with PLGA and cast (150 µm) on Mo foil (30 µm). A polyanhydride layer on top of a PLGA layer was used to encapsulate the battery after fabrication. The battery featured a lifetime of about 13 days and a total energy capacity of 6.5 mW h cm^−2^, when discharged at a current density of 25 µA cm^−2^, with an output voltage around 1.5 V for 50 h, during which molybdenum oxide was reduced and dissolved, and 0.6 V during the next 250 h, during which molybdenum foil acted as cathode active material. Remarkably, the high voltage output during the first phase was maintained up to a discharge current density of 150 µA cm^−2^. As proof‐of‐concept applications, the battery was used to power a digital calculator and a low‐power ECG signal detector. Powering of a standard LED over 16 h upon immersion in PBS was also demonstrated. Degradation of the battery (with magnesium and molybdenum foil thicknesses reduced to 50 and 5 µm, respectively) was investigated in PBS: at 37 °C, the polyanhydride and PLGA encapsulation degraded first, followed by the simultaneous dissolution of Mg, sodium alginate hydrogel, MoO_3_/PLGA layer, and Mo. Most materials completely dissolved within 9 days, except Mo that needed further 10 days at 85 °C to fully dissolve. In vitro cytotoxicity tests of MoO_3_ paste electrode encapsulated by a PLGA layer showed a good biocompatibility after co‐incubation with L‐929 mouse fibroblast cells. Further, in vivo subcutaneous implants (using a battery with reduced foil thickness) performed on a rat model demonstrated full degradability of the entire battery in 4 weeks, without apparent inflammatory response on skin tissues and on different organs.

All the bioresorbable batteries discussed above are rather bulky compared to other electrical (and optical) bioresorbable components; further, their performance quickly and continuously degrade when operated in biofluids, so that energy provided should be used in a short time to limit losses, while complete dissolution of battery materials require a much longer time.

#### 3.3.2 Energy Harvesters

**Figure 9:**
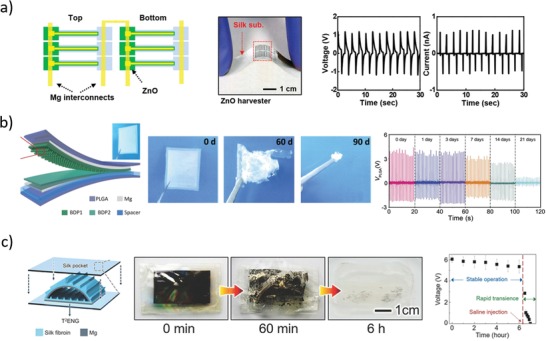
Representative examples of mechanical energy harvesters. a) Left: Sketch of the ZnO piezoelectric energy harvester.^73^ Middle: Photograph of a fabricated device, demonstrating flexibility on a silk substrate. Right: Device electrical output during periodic bending. Reproduced with permission.^73^ Copyright 2013, John Wiley and Sons. b) Left: Sketch and photograph (inset) of the polymeric triboelectric energy harvester.^111^ Middle: Representative photographs of device dissolution during immersion in PBS at 37 °C. Right: Electrical output degradation during in vivo implantation in rat. Reproduced with permission.^111^ Copyright 2016, The Authors, Published by American Association for the Advancement of Science. c) Left: Sketch of the optically monitorable silk triboelectric energy harvester.^112^ Middle: Photograph of in vitro device degradation during immersion in DIW at room temperature. Right: In vivo demonstration of device degradation after subcutaneous injection of physiological saline solution. Reproduced with permission.^112^ Copyright 2018, John Wiley and Sons.

Energy harvester devices, such as piezoelectric^109^ and triboelectric^110^ generators, able to directly and continuously produce energy once implanted in human body, represent an appealing alternative to energy storage devices, such as, batteries (**Figure** **9**).

Dagdeviren et al.^73^ reported a mechanical energy harvester using zinc oxide (ZnO) as piezoelectric material deposited on a silk substrate (Figure 9a). ZnO strips (500 nm thick) were provided with top and bottom magnesium electrodes (500 and 300 nm thick, respectively) defining an active area of 50 µm × 2 mm. The energy harvester consisted of 60 single Mg/ZnO elements connected in series and parallel, namely, six series groups of ten parallel elements. Application and release of buckling stimuli to the silk substrate led to bending of the ZnO strips, resulting in positive and negative variations of voltage and current outputs. Peak values of about 1.14 V and 0.55 nA were achieved, respectively, with a peak output power density of 10 nW cm^−2^. Dissolution tests were carried out in deionized water, PBS, and serum at room temperature. The silk substrate (about 25 µm thick for this case study) quickly dissolved in water, causing disintegration of the device physical structure. Afterward, all electronic materials, i.e., Mg, MgO, and ZnO, completely dissolved in 15 h in a controlled manner, without cracking, flaking, or delamination.

Triboelectric effect, differently from piezoelectric effect, relies on different charge affinity of two (usually dielectric) materials. When in contact (or even rubbed against each other), electron clouds overlap, and there is an electron redistribution at the two material interfaces. After separation, most of the electrons remain trapped in the new potential wells, resulting in surface polarization of the two materials, that now have an excess and a vacancy of electrons, causing negative and positive interface charge, respectively. This surface charges can produce current flow when the materials (contacted to an electric load) are displaced one respect to each other, being the system equivalent to a capacitor with varying capacitance.^110^

Zheng et al.^111^ fabricated biodegradable triboelectric nanogenerators using PLGA and PCL films featuring a nanostructured surface (Figure 9b). The nanogenerator had a multilayered structure. PLGA and PCL slabs (2 cm × 3 cm, 50–100 µm thick) were patterned at the nanoscale on one surface to increase effective contact area, and the two polymer layers were assembled facing each other as friction parts, with a 200 µm thick spacer set between them. A magnesium film (50 µm thick) was then deposited on the back flat side of each friction layer as electrode, and the whole structure was eventually encapsulated in PLGA (75:25 lactic:glycolic ratio, 100 µm). Upon application of a periodic compression force (and subsequent release) at a frequency of 1 Hz to simulate low‐frequency biomechanical motion, a periodic output signal with peak voltage of about 40 V and peak current of about 1 µA was achieved. By connecting the nanogenerator to a load resistance of 80 MΩ, a power density of 3.26 µW cm^−2^ was achieved. As a proof‐of‐concept application, primary neurons were repeatedly (at 1 Hz, 10 V mm^−1^) stimulated with the nanogenerator for 5 days, after 24 h of initial culture, showing that the nerve cell growth was successfully orientated, which was crucial for neural repair. In vitro degradation experiments were performed in PBS at pH 7.4 and 37 °C, showing slow water uptake of PLGA in the first month, followed by a rather rapid swelling and mass loss that led to structural failure of PLGA after 50 days, and, in turn, to complete dissolution of the whole device after 90 days. The biocompatibility of the nanogenerator was assessed by culturing endotheliocytes (ECs) on both PLGA and PCL films (≈100 µm). After 7 days, most of the ECs were viable and showed no significant difference with the reference control group (standard cell culture). In vivo testing was carried out by subcutaneous implantation of the nanogenerator in the dorsal region of a rat, and monitoring the output voltage of the implanted nanogenerator. The output voltage (generated upon finger‐tapping stimulation through skin) was stable for one week, then slowly decayed until complete failure after one month. On the other hand, after 9 weeks from implant, the wound was healed with no infection or inflammation signs observed, and the integrity of the device structure had been destroyed, indicating that most of the materials were biodegraded in the animal body.

Recently, Zhang et al.^112^ reported a silk‐based triboelectric generator in which the degradation state can be optically monitored (Figure 9c). Magnesium and silk, which was longitudinally patterned at the microscale, were used as triboelectric materials. The Mg film also functioned as electrode. Silk with different thickness and crystallinity was eventually used as encapsulation material. This silk micropattern had the twofold aim of increasing the triboelectric response and creating a diffractive grating operating in the near infrared (NIR) spectral region (at 1040 nm). Illumination of the triboelectric generator with a laser at 1040 nm produced a diffraction pattern that smoothened and eventually vanished together with the triboelectric response of the generator, as the micropattern structural integrity degraded due to silk dissolution. In vitro characterization of the generator was carried out through application of mechanical impulses at a frequency of 2 Hz to mimic low‐frequency biomechanical motions. An open‐circuit voltage up to about 60 V and a short‐circuit current (absolute value) as high as 1 µA were achieved, depending on the load resistance. A power density of about 3.85 µW cm^−2^ was recorded using a load resistance of 100 MΩ. Remarkably, by using different thicknesses and crystallinity degrees for the encapsulating silk, the device lifetime in water was tuned from tens of minutes (unencapsulated) to over 10 h (encapsulated in a double layer of 50 µm thick crystallized silk), with failure being caused by magnesium dissolution after destruction of the encapsulating material. The triboelectric signals and diffractive optical readouts (first‐order diffraction intensities of the silk micrograting monitored at 405, 532, and 650 nm) showed highly positive correlation with the device degradation (in vitro). In vivo experiments were carried out on encapsulated generators implanted in the subdermal region of mice. The implanted generator showed a stable output of about 6 V for 6 h, when a constant external force was repeatedly applied onto the skin of the implanted region. Injection of 10 mL of physiological saline solution at the implant region was used to trigger degradation, with triboelectric function being lost within 30 min. The implant region was reexamined after three weeks, showing gradual reintegration and apparent revascularization in the subdermal layers with no obvious inflammatory reactions detected, so proving good in vivo compatibility of the device. Tests carried out with silk components crystallized to a lower degree (water vapor annealing for 2 h) completely disappeared after three weeks in vivo, whereas for a similar device with a higher crystallization degree (water vapor annealing for 12 h) residues were still observed in the implant region after three weeks. Finally, as a proof‐of‐concept application, the authors loaded penicillin or phenobarbital within the silk encapsulation and/or triboelectric layer and demonstrated in vivo drug release in an animal model. Penicillin release was tested in rats infected at the surgical site with *Staphylococcus aureus*. Drug loading had negligible effect on the electrical properties of the generator and did not affect silk degradation. Analysis of the infected tissue and bacterial culture on infected samples revealed that infection was healed at day 7 from implantation. The device was also used as epilepsy sensor/medicament by analyzing (with an external unit) the output amplitude and frequency of a phenobarbital‐loaded device. When epileptic symptoms were induced in anesthetized mouse with Penicillin G Sodium injection, the control unit recognized the pathologic state and activated a resistor in order to accelerate silk dissolution and thus increase phenobarbital release: after a 10 min treatment, the epileptic convulsion was remarkably alleviated.

Photovoltaic (PV) cells^113^ represent an alternative choice to piezoelectric and triboelectric generators for in vivo energy harvesting. Lu et al.^114^ reported on a bioresorbable array of photovoltaic microcells that were fabricated using biocompatible and biodegradable materials. The single element of the array of microcells consisted of a thin p–n Si photodiode (thickness 1.5 µm, dimensions 390 µm × 410 µm, B and P concentrations about 10^20^ cm^−3^, back‐side surface coated with a SiO_2_ layer), with patterned Mo (thickness 1.5 µm) serving as electrodes and for electrical interconnections; PLGA (200 µm thick) was used as both bioresorbable substrate and encapsulation coating. The single element (with a fill factor of about 73%) open‐circuit voltage and short‐circuit current were 0.40 V and 4.34 mA cm^−2^, respectively, when exposed to 1 sun illumination at room temperature (solar simulator with AM 1.5G filter and 100 mW cm^−2^ power density). The whole array of photovoltaic microcells (72 in total), connected as 12 columns in series and 6 rows in parallel, resulted in open‐circuit voltage and short‐circuit current of 4.84 V and 34.45 mA cm^−2^, respectively, for a total power of 122 µW (enough to power a commercial blue LED). In vitro dissolution tests of the array of microcells carried out in PBS at pH 7.4 and 37 °C showed device failure after 5 days from immersion, mainly due to dissolution and water penetration through the PLGA encapsulation layer, whereas complete dissolution of the device required 1–2 months. Ex vivo functional tests were performed with porcine skin (with and without fat), showing degraded performance of the array of microcells due to significant skin absorption in the UV–vis range (transmission without fat about 20% up to 500 nm, and about 60% beyond 800 nm). Placing fat under the skin contributed to an additional transmittance loss up to 25% for wavelengths between 400 and 1100 nm. For instance, upon illumination with a NIR LED (780 nm, 200 mW cm^−2^) under 2 mm thick porcine skin with 2 mm thick fat underneath, a total power of 64.4 µW was reported. In vitro cytotoxicity was investigated (immunofluorescence live/dead assay) with human umbilical vein endothelial cells (HUVECs) seeded and cultured for 7 days on photovoltaic microcell array as substrate (dimension 2000 µm × 4000 µm × 1.5 µm), revealing good biocompatibility. Eventually, in vivo tests were carried out by implanting a photovoltaic microcell array (used to power an external blue LED) in the infrascapular region of an adult Sprague–Dawley rat, showing operation (under NIR irradiation) of the implanted device for 3 days. During 4 months from implantation, the rat showed no signs of disease or debilitation, and little/no device residues were apparent under visual and optical microscope (H&E staining) evaluation after this time.

## 4 Bioresorbable Optical Devices

**Figure 10:**
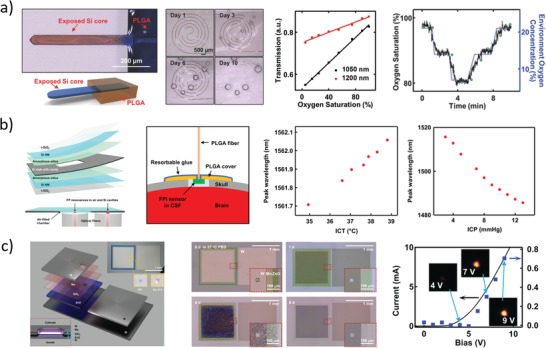
Representative examples of optical devices. a) Left: Photograph and sketch of a bioresorbable silicon waveguide.^123^ Middle‐Left: Representative photographs of a spiral waveguide dissolution during immersion in PBS at 70 °C. Middle‐Right: In vitro calibration of the optical transmittance for oxygen concentration measurement. Right: Comparison between the measured oxygen saturation and the environmental concentration. Reproduced with permission.^123^ Copyright 2018, John Wiley and Sons. b) Left: Sketch of the structure and functioning principle of the optical pressure sensor.^124^ Middle‐Left: Sketch of the implanted device configuration. Middle‐Right: In vivo calibration of the temperature sensor, against commercial sensor measurements. Right: In vivo calibration of the pressure sensor, against commercial sensor measurements. Reproduced under the terms of the CC BY license.^124^ Copyright 2019, The Authors, Published by American Association for the Advancement of Science. c) Left: Sketch and optical microscope images of the bioresorbable ZnO LED.^36^ Middle: Optical microscope images during dissolution of the bioresorbable LED in PBS at pH 7.4 and 37 °C. Right: Electrical characteristic of the ZnO LED; insets: optical microscope images showing device electroluminescence. Reproduced with permission.^36^ Copyright 2019, John Wiley and Sons.

Absorption and scattering of light in tissues result from fundamental light–matter interactions and have enabled a variety of stimulation and monitoring techniques for biomedical therapy and imaging.^115^ Very recently, bioresorbable optical waveguides, photonic structures, and optical sensors working in the vis–NIR spectral region and able to operate in vivo in physiological conditions have also attracted attention for photomedicine applications^116^, ^117^, ^118^ (**Figure** **10**).

In 2016, Nizamoglu et al.^119^ reported on comb‐shaped bioresorbable waveguides fabricated using silk, poly(vinylpyrrolidone) (PVP), PLLA, and PLGA, which were able to deliver light into living systems down to a depth >10 mm. The thickness of the films was typically adjusted in the range 200–800 µm to achieve a suitable waveguide cross‐section so as to optimize mechanical rigidity/flexibility and optical extraction efficiency, depending on the targeted depth and, in turn, specific application. For instance, PLLA waveguides with cross‐section of 240 × 650 µm^2^ featured loss coefficients of 0.16 dB mm^−1^ in air, 0.76 dB mm^−1^ in water, and 2 dB mm^−1^ in oil, which were adequate for applications where the majority (90%) of input optical energy has to be delivered through a tissue section down to depth of 10 mm. By increasing the cross‐section to 440 × 580 µm^2^, loss coefficients reduced to 0.15 dB mm^−1^ in air, 0.62 dB mm^−1^ in water, and 1.5 dB mm^−1^ in oil. In vitro dissolution experiments of different polymer waveguides were carried out in PBS over 24 h: PVP waveguides dissolved within minutes; silk deformed after several minutes and swelled within hours, but did not fully dissolve; PLGA and PLLA largely retained their physical structure, with modest degradation of the optical transparency over 24 h. To assess biodegradation of polymer waveguides in vivo, transparent pieces of PLGA 50:50 (1 × 5 × 0.5 mm) were subcutaneously implanted in living mice for 35 days. Shape and transparency of the implant were largely intact at day 6, though significant degradation of both shape and transparency was apparent at day 17, and on day 35 the implant was not visible to naked eye. Visual inspection and histology indicated no signs of inflammation at the implantation site, proving good biocompatibility. In situ waveguide‐assisted photochemical tissue bonding (PTB), which is a dye‐assisted photochemical technique that induces crosslinking between wound surfaces for skin wound enclosure, was demonstrated in animals, using a device with three PLA waveguides to deliver light to porcine skin tissues. Each waveguide was tapered, with a uniform region of 1 mm in width, 440 µm in thickness, and 10 mm in length, and had corrugated edges for optimal extraction of optical energy over the entire length of 10 mm. A full‐thickness incision was made on the excised dorsal skin of a pig immediately after being killed, and Rose Bengal dye was applied to the wound. The polymer waveguides were inserted into the wound, with tissue sides brought in physical contact with the waveguide surface, and a 532 nm laser light at 1 W was launched for 15 min. Eventually, the protruding polymer was trimmed, leaving the comb teeth inside the wound to freely degrade. Tensiometer‐based shear tensile strength measurements showed that the wound treated with the bioresorbable waveguides had a shear strength six times higher than wounds treated with conventional surface‐illuminated PTB (i.e., 1.94 kPa vs 0.33 kPa).

In 2018, Fu et al.^120^ reported PLLA optical fibers to be used as a bioresorbable optical neural interconnection between biological matter and optical instrumentation, thanks to negligible optical losses and high transmission coefficient (about 95%) of amorphous PLLA in the visible range.^121^, ^122^ The fiber diameter was tuned to about 220 µm, like that of standard silica optical fibers, to facilitate interconnection with commercial optical components. PLLA fibers exhibited a bending stiffness of about 1.5 × 10^4^ N m^−1^, which is about ten times smaller than that of conventional silica fibers with same geometry (stiffness of 2.4 × 10^5^ N m^−1^) and thus ensured higher flexibility for in vivo biomedical applications. In vitro dissolution experiments were performed in PBS at pH 7.4 and room temperature for 42 days, during which fiber propagation losses were evaluated. Surface erosion of PLLA fibers occurred with soaking in PBS, resulting in increased light scattering and, in turn, augmented propagation losses (loss coefficient of 1.64 dB cm^−1^ at day 0, and 4.9 dB cm^−1^ at day 42). Accelerated dissolution experiments were performed in vivo using fibers made of PLGA 50:50 (with shorter degradation time, compared to pure PLLA that required years for complete dissolution). The fibers were implanted in the brain of different three‐month‐old mice at a depth of 2.5 mm (covering the whole depth of cortex), and perfusion and brain section were performed after different days. On the day of implantation, the lesion created by the PLGA fiber was almost equivalent to that of a silica fiber with similar geometry. As the PLGA fiber gradually and fully dissolved in the brain over the two weeks following implantation, the lesion in the brain region was reduced, and almost disappeared on day 60. Further PLLA fibers were applied for in vivo brain function investigation, including neural signal sensing and interrogation. The PLLA fibers were implanted in the brain of mice and connected to standard optical setups for photometric and optogenetic experiments, namely, enhanced green fluorescence protein recording in deep‐brain hypothalamus, and optogenetical stimulation of hippocampus neurons with a blue laser (at 473 nm) to induce seizures. In both cases the fibers were fully functional at day 0 and gradually decreased their performance until functionality ceased around day 10, though PLLA fibers were not fully dissolved after 10–15 days. However, tissue damage induced at the implantation region was fully recovered after 60 days, suggesting a promising pathway for neural activity research. The latter was evaluated through immunohistochemical staining of neurons using NeuN, a red biomarker for neurons.

Bai et al.^123^ fabricated flexible infrared waveguides using thin filaments of Si NMs to tackle major drawbacks of PLGA and PLLA waveguides, such as, susceptibility to swelling as a result of water uptake, and limited refraction index contrast, resulting in increased propagation losses and poor optical mode confinement in tissues and biofluids (Figure 10a). Flexible silicon filaments were obtained from SOI wafers by patterning silicon nanomembranes to form planar coiled structures, for instance, zigzag and spiral waveguides, which were then transfer‐printed on and coated with a PLGA layer (10 µm thick). Eventually, laser milling was used to define strips of PLGA acting as cladding, surrounding the Si filaments, acting as core. The coiled silicon waveguides were designed to withstand an unfurling strain up to 99% without breaking. Optical losses associated with unfurling of the waveguides were reported to be negligible (around 0.05 dB cm^−1^), compared with optical losses associated with absorption in the PLGA cladding and scattering from imperfections in the silicon filamentary structures (around 0.7 dB cm^−1^ in total). Accelerated dissolution experiments of the waveguides (Si core with thickness of 1500 nm and width of 50 µm, PLGA cladding with thickness of 10 µm) were performed in PBS at pH 7.4 and 70 °C. Transmitted power steadily decreased over time, until (at day 6) losses prevented waveguiding, in agreement with dramatic decrease in transparency of PLGA by day 3, which was consistent with swelling, water uptake, and initial stages of hydrolysis, and consequent dissolution of the silicon core, which was fully degraded by day 10. By selectively removing the PLGA cladding in a local region of the waveguide, the silicon core was exposed to the surrounding medium, thus acting as a transient optical sensor. The Si/PLGA transient optical sensor was used for glucose sensing in mouse blood in vitro, monitoring the return losses at 1160 and 1330 nm in the concentration range 120–180 mg dL^−1^ (glucose blood concentration of interest in humans is 70–180 mg dL^−1^). Calibration curves exhibited a good linearity in the investigated glucose concentration range, though interfering effects associated with absorption by other biological species were not considered. The transient optical sensor was further used in vitro and in vivo to measure blood oxygen saturation by injecting light in the waveguides (at one end) at 1050 and 1200 nm, and measuring light transmitted through the waveguide (at the other end) at those wavelengths (corresponding to absorption at 1050 and 1200 nm). In vitro tests of hemoglobin in PBS at various oxygenation levels showed calibration curves with a linear response from 5% to 100% oxygen saturation. In vivo experiments were carried out by inserting the transient optical sensor into the subcutaneous region of mice near the thoracic spine and connecting the fibers to external light sources and optical power meters. The experiments involved changing the concentration of oxygen in the experiment chamber to trigger corresponding changes in the oxygen saturation of the blood hemoglobin (SO_2_). The oxygen saturation measured by the transient optical sensor was consistent with the measurement of a commercial oximeter positioned at the paw of the mice, with uncertainty of about 4% SO_2_, mostly arising from motion artifacts, tissue heterogeneities, and venous pulsations. The implanted transient optical sensor was stained with tungsten nanoparticles in order to monitor its dissolution in vivo through microcomputed X‐ray tomography (microCT) imaging, highlighting complete degradation after 15 days.

In 2019, Shin et al.^124^ reported on bioresorbable optical sensors based on resonant effects, for instance, a Fabry–Pérot interferometer (FPI) and a photonic crystal cavity, for in vivo pressure and temperature measurements (Figure 10b). Concerning the FPI, starting with SOI wafers, an air cavity was realized as a square through‐hole (250 × 250 µm size) in a Si slab (10 µm thick), which was sandwiched between two Si NM (250 nm thick) diaphragms, using amorphous silica (200 nm thick, obtained from PDMS calcination) as an adhesion layer; thermal SiO_2_ (10 nm thick) was used as encapsulation layer. Bioresorbable PLGA (75:25 lactic:glycolic ratio) optical fibers with diameter of 200 nm were used to couple the FPI with commercial optical sources/spectrometers for both pressure and temperature measurements, by monitoring the infrared spectral region around 1500 nm. For pressure sensing, the optical fiber was coupled with the air cavity region of the FPI, whereas for temperature sensing it was coupled with the noncavity (solid) region of the FPI. Pressure‐induced deflections of Si NM diaphragms changed the optical thickness of the air cavity, causing shifts (toward shorter wavelengths) of the position of Fabry–Pérot resonant peaks in the reflection spectrum; temperature‐dependent changes of the refractive index of Si (positive thermo‐optic coefficient) changed the optical path of light traveling within solid Si layer of the FPI and induced, again, shifts of the position of Fabry–Pérot resonant peaks in the reflection spectrum. Experimental results achieved on FPI sensors resulted in a sensitivity of −3.8 nm mmHg^−1^ and an accuracy of ± 0.40 mmHg in the range 0–15 mmHg for pressure sensing, and a sensitivity of 0.090 nm °C^−1^ in the range 27–46 °C for temperature sensing. In vitro degradation tests of FPI in PBS at pH 7.4 and 95 °C showed a complete dissolution of the device within 80 h (corresponding to about 195 days at 37 °C). The bioresorbable fiber performance (i.e., transmission efficiency) reduced in 3 days when immersed in PBS at 37 °C, due to polymer swelling upon water uptake, and were fully degraded over a period of 3 weeks. Operation lifetime of the FPI was tested in PBS at 37 °C over a period of 8 days using a device with thicker thermal SiO_2_ encapsulation layer (thickness 300–1000 nm) and air cavity (thickness 100 µm). A highly stable pressure response throughout the test period (variation within ± 6%) was observed, while that obtained from a device without SiO_2_ layer (air cavity thickness 10 µm) under similar test conditions exhibited a gradual increase in sensitivity and baseline over time. In vivo experiments were carried out with FPI sensors implanted in rat skull, successfully measuring intracranial pressures in the range 3–13 mmHg (sensitivity −3.1 nm mmHg^−1^, around 1518 nm) and temperatures in the range 34.9–38.8 °C (sensitivity 0.089 nm °C^−1^, around 1571.7 nm), with sensing performance that was in good agreement with that achieved in vitro. Eventually, histology of brain, heart, kidney, liver, lung, and spleen tissues collected from animals with an FPI device implanted in the brain after 5 weeks was conducted; the results were comparable with those achieved from a control mouse without any implant. Histopathological evaluation of the acquired images revealed no signs of inflammation, necrosis, or structural abnormality in any organ.

Very recently, Lu et al.^36^ demonstrated a fully bioresorbable LED based on a II–VI semiconductor, namely, ZnO, and an ultrathin transparent electrode of Mo (Figure 10c), advancing the state‐of‐the‐art research on this subject, with respect to partially biodegradable riboflavin‐ and peptide‐based organic LEDs,^125^, ^126^ and demonstrating full biodegradability of the two key elements of a LED, namely, direct bandgap semiconductor that allows recombination of injected electrons and holes for light emission, and transparent electrode that allows escaping of the emitted light. Specifically, a ZnO layer (200 nm thick) used as n‐type semiconductor was deposited on top of a Si (12 µm thick) membrane used as p‐type semiconductor, whereas W (100 nm) and Mo (8 nm) were used for electrical contacts. The LED emitted light starting from a threshold voltage of about 5 V, with a rather broad emission spectrum ranging from 420 to 650 nm and a maximum optical power density of 0.7 mW cm^−2^, at 9 V (maximum voltage before device damage). Although the threshold voltage was comparable to that of standard ZnO p–n junction LEDs,^127^, ^128^ the intensity of the emitted light was significantly lower. Addition of bioresorbable Fabry–Pérot optical filters based on silicon nanomembranes was proposed to select specific wavelength bands in the visible region and narrow, in turn, the emission spectrum. Dissolution tests were performed in vitro in PBS at pH 7.4 and 37° C, showing dissolution of thin Mo within 1 day, whereas ZnO and SiO_2_ disappeared in 8 and 30 days, respectively, and W degradation occurred in 80–200 days. Full degradation of the Si substrate (12 µm tick) under physiological conditions was estimated to occur in about 5 years, though this time can be reduced by decreasing the Si thickness. No in vivo tests were reported for the proposed LED.

## 5 Implanted Bioresorbable Systems

The ultimate goal of bioresorbable devices is their synergistic integration into an autonomous system to be implanted in the human body, able to monitor parameters of clinical interest, and then degrade on‐demand once no more needed. In spite of the significant number of works in which bioresorbable components have been tested in vivo, only in a few cases two or more bioresorbable components belonging to different functional subsystems (sensor/transducer, electrical/optical readout, power/driving) were interconnected into an implantable and bioresorbable system.

In this section, we will review the effort paid over the last decade on the development of implantable bioresorbable opto‐electronic systems, intended as interconnection of two (or more) different bioresorbable functional subsystems. A summary of the bioresorbable devices and systems tested in vivo is reported in **Table** **1**.

**Table 1:** Summary of principal in vivo experiments with bioresorbable devices and systems

In vivo experiments carried out with bioresorbable components and systems		
Bioresorbable device	In vivo test	Ref.
Si‐based transistors with Au electrical contacts on silk substrate.	Subcutaneous implantation in mouse for biodegradation and biocompatibility	65
Discrete Mg and Si‐based circuitry elements, i.e., capacitors, inductors, resistors, diodes, and transistors. Mg antenna with MgO protection on silk substrate and with silk encapsulation. Mg coil and Si serpentine with MgO insulation on silk substrate and with silk encapsulation.	Subcutaneous implantation in rodents for biodegradation and biocompatibility. Subcutaneous implantation in rat, with power transfer functionality tests for 15 days. Subcutaneous implantation in rat and wireless power transfer for thermal therapy.	28
Silicon transistors with Mg electrical contacts on silk substrate.	Subcutaneous implantation in mouse for biodegradation and biocompatibility	68
Mg resistor on levan polysaccharide.	Subcutaneous implantation in mouse back, and irradiation with NIR laser to accelerate degradation.	71
Mg coil and resistor on silk fibroin, with possible antibiotic loading.	Subcutaneous implantation in rats for wireless power transfer for thermal therapy and drug delivery.	81
Mo coil and resistor on PLGA substrate, with doxorubicin‐loaded lipid membrane.	Subcutaneous implantation in rat for biodegradation and biocompatibility. Subcutaneous implantation in porcine model and on‐demand drug release.	82
Mg coil and capacitor, Mg/Mo electrodes, and Si diode on PLGA substrate.	Implantation in rat and electrical nerve stimulation for 10 min every day for 6 days.	83
Si NMs on PLGA sealed on nanoporous Si substrate, with SiO_2_ passivation and polyanhydride encapsulation.	Implantation in rat brain, for intracranial pressure (3 days) and temperature (6 days) measurement.	94
Si NMs on amorphous SiO_2_ suspended membrane on Si substrate; encapsulation with SiO_2_.	Implantation in rat brain, for intracranial pressure and temperature measurements for 18 days, with complete signal loss after 25 days.	95
Thermo‐mechanically treated PLLA piezoelectric sensor with Mg and Mo electrical connections.	Implantation in rat: subcutaneous for bioresorption, under diaphragm to demonstrate force measurement.	96
Mg electrodes on PLLA substrate, with PGS spacer, and POMaC + PGS encapsulation.	Subcutaneous implantation in rat, for pressure and strain measurements over 24 days.	91
Mg traces with PGS and PLGA spacers on POMaC and PHB/PHV substrates.	Implantation around rat femoral artery for wireless heart rate measurement for one week.	92
Si NMs with SiO_2_ encapsulation on PLGA substrate. Similar Si transistors‐multiplexed Mo electrode array.	Implantation in rat brain for ECoG measurement over one month. Spatiotemporal mapping of evoked potentials in rat brain.	101
Si NMs with Mo connections with SiO_2_ encapsulation on PLGA substrate.	Implantation in rat brain for ECoG and evoked potential recording.	33
Battery with Mg anode, Mo/MoO_3_ in PLGA paste cathode, polyanhydride encapsulation.	Subcutaneous implantation in mouse for biodegradation and biocompatibility.	108
Triboelectric generator in PLGA and PCL with sputtered Mg, PLGA encapsulation.	Subcutaneous implant in rat, finger‐tapped every day for one month for performance monitoring.	111
Silk and Mg triboelectric generator with silk separator.	Subcutaneous implantation in rat, performance measurement for 6 h and stimulated degradation.	112
Si NM photodiodes with SiO_2_ encapsulation and Mo interconnections on PLGA substrate.	Subcutaneous implantation in rat, with photovoltaic cells functioning for 3 days supplying a blue LED.	114
PLGA and PVP waveguides.	Subcutaneous implantation in rat for biodegradation.	119
PLGA and PLLA optical fibers.	Implantation in rat brain for biodegradation, fluorescence measurement and optogenetics.	120
Si waveguide on PLGA substrate.	Subcutaneous implantation in rat for oxygen saturation measured by NIR absorption.	123
Si NMs on amorphous SiO_2_ suspended membranes on Si substrate, SiO_2_ encapsulation and PLGA optical fiber.	Implantation in rat brain, for acute intracranial pressure and temperature measurements.	124
PLGA fiber on Si photodiodes with Zn connections on PLGA substrate; encapsulation with SiO2.	Implantation in mouse brain, for intracranial temperature and oxygenation measurements; proof‐of‐concept of brain calcium concentration measurement.	132
Mg‐Zn‐Mn alloy (ZM21) stent loaded with CeO_2_ and Au nanoparticles.	Insertion in dog carotid, demonstrating digital data transfer, reduced immune response and NIR and RF‐induced thermal therapy.	129

**Figure 11:**
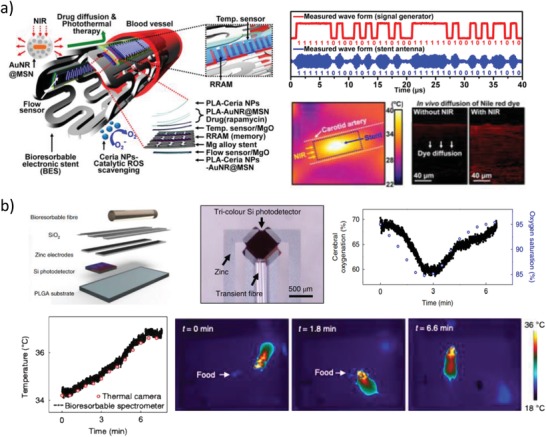
In vivo bioresorbable implanted systems. a) Left: Sketch of the multifunctional bioresorbable stent.^129^ Right‐Top: Wireless data transmission between an external antenna and the implanted stent. Right‐Bottom: Thermal camera image of the Au@MSN‐covered implanted stent during NIR irradiation (left) and in vivo thermal‐assisted Nile Red diffusion (right). Reproduced with permission.^129^ Copyright 2015, American Chemical Society. b) Top‐Left: Sketch of the structure of the bioresorbable fiber‐photodetector system.^132^ Top‐Middle: Optical microscope image of a fabricated device with RGB detector. Top‐Right: In vivo response to brain oxygenation change of the red photodiode, and comparison with tail blood oxygenation measurements. Bottom‐Left: In vivo response of the bioresorbable intracranial temperature sensor, and comparison with thermographic camera measurements. Bottom‐Right: Thermographic camera images of freely moving rat during food searching and eating. Reproduced with permission.^132^ Copyright 2019, Springer Nature.

A bioresorbable multifunctional stent was proposed by Son et al.,^129^ capable of measuring flow and temperature, store and wirelessly transmit recorded data, and release drugs upon stimuli (**Figure** **11**a). A standard bioresorbable magnesium‐zinc‐manganese alloy (ZM21) was used as stent mesh material and RF antenna, and acted as substrate for all the others components, namely a resistive RAM (RRAM),^130^, ^131^ a resistive temperature sensor, and a thermoresistive flow sensor. Therapeutic effects were also designed with gold nanoparticles for RF‐ and NIR‐triggered drug release, and cerium oxide nanoparticles as anti‐inflammatory agent. A magnesium serpentine (100 nm thick, with 400 nm MgO encapsulation and 15 µm PLA) served as thermoresistive sensor, with a thermal coefficient of about 0.08% °C^−1^ (calibrated in vitro), and as flow sensor, by monitoring serpentine resistance change with time. An MgO (12 nm thick) active layer sandwiched between two Mg electrodes (60 nm thick) was used for the fabrication of the RRAM, with set and reset voltages of −0.8 and +0.7 V, respectively, and an estimated retention time of several years.

Ex vivo testing on canine aorta of a two‐bits RRAM coupled to the flow sensor demonstrated the possibility of measuring and storing data on the same device (data analysis and clustering, power supplying and memory driving were performed by an external PC). CeO_2_ nanoparticles for reactive oxygen species (ROS) scavenging were englobed in PLA encapsulating layer, to act as catalyst and reduce the concentration of ROS that promote apoptosis ant restenosis. CeO_2_ nanoparticles were tested in vitro with HUVECs and cardiac muscle cells (HL‐1), showing cell viability preservation already after 15 min exposure to 50 × 10^−6^ m H_2_O_2_, while in control experiments without CeO_2_ nanoparticles cell viability dropped to 55% and nearly 0% for HUVECs and HL‐1 cells, respectively. Gold nanoparticles with mesoporous silica shell (Au@MSN) were used to thermally trigger drug release via RF signal or NIR laser exposure. In vivo tests were performed by deploying the bioresorbable stent with CeO_2_ and Au@MSN nanoparticles‐soaked PLA encapsulating layer in canine aorta: Nile Red dye was used as drug model to demonstrate increased tissue permeability enabled by Au@MSN heating induced by NIR laser and/or RF coil stimulation, while CeO_2_ nanoparticles inhibited macrophage migration and inflammatory response. Moreover, data transmission at 1 Mbps using the stent structure as an RF antenna was successfully demonstrated in vivo (though the stent was externally wired to the network analyzer), with a power transfer efficiency of −21.8 dB at 900 MHz, at a working distance of 1 cm. Although Au@MSN and CeO_2_ nanoparticles were not bioresorbable, injection into an 8‐weeks‐old mouse showed complete clearance after 50 h, with no adverse effects on the animal. No biodegradation experiments were reported for the device.

Very recently, a bioresorbable optoelectronic system was reported by Bai et al.,^132^ consisting of an optical fiber used to deliver light, a photodetector for generating electrical signals in response to transmitted light, and electrodes for electrical interconnection of the photodetector to external measurement setups (Figure 11b). The system was assembled into a shape that resembled hypodermic needles (600 µm wide, 160 µm thick, and several mm long) to facilitate minimally invasive implantation. The optical fiber consisted of a 150 µm diameter PLGA (75:25 lactide:glycolide ratio) core coated with an alginate hydrogel serving as a cladding. The photodetector was made from crystalline Si NMs transfer‐printed on a PLGA substrate (10 µm thick) in two different configurations: interdigitated p‐i‐n junctions (1500 nm thickness), and tri‐color stacked junctions (four highly doped silicon layers with different thicknesses and alternating doping, namely, 200 nm (n^+^‐doped), 400 nm (p^+^‐doped), 1400 nm (n^+^‐doped), and 4500 nm (p^+^‐doped)). Metal electrodes for readout of the photodetector were made of zinc (thickness 400 nm). Eventually, the photodetector provided with electrodes was encapsulated with sputtered SiO_2_ (50 nm). The optical fiber (connected to a commercial laser source) was then placed on top of the 10 µm thick PLGA substrate containing the photodetector/electrode, through the 50 nm thick SiO_2_ encapsulating layer. An additional bioresorbable optical filter (distributed Bragg reflectors) made of a multistack of alternating layers of SiO*_x_* and SiN*_x_* with controlled thickness and periodicity was also reported, to be placed on top of the photodetector with the aim of filtering out laser excitation and/or collect fluorescence emission. A simple bioresorbable spectrometer was demonstrated using the tri‐color photodetector coupled with a PLGA fiber for light delivery, and the four Zn metal electrodes for electric readout. *I*–*V* curves (in a dark environment) for the three junctions of the photodetector indicated excellent rectifying behaviors, with dark currents of about 10^−1^–10^−2^ µA and responsivities of about 0.15 A W^−1^. In vitro biodegradation experiments of the system encapsulated with a 200 nm thick SiO_2_ layer were performed in PBS at 37 °C, resulting in stable operation for 10 days, followed by performance degradation (in terms of *I*–*V* curves and responsivity of the photodetectors), until at day 25 the system ceased functioning. Computed X‐ray tomography images of the system implanted in the subcutaneous region near the flank of a mouse model showed that different materials and components were bioresorbed at different times, with complete bioresorption achieved on day 45. Further hematology and biochemistry studies were carried out to infer into the biodistribution of elements (Si and Zn, associated with dissolution of the biodegradable spectrometers implanted in mouse models) in blood, brain, heart, kidney, liver, lung, muscle, and spleen tissues explanted from mice at 1, 3, 5, and 7 weeks after implantation. The results in all the measured organs of mice with an implanted device, compared with those in the control group with no implantation, showed no abnormal accumulation of dissolved Si and Zn in the tissues during the 7‐week‐long implantation period. Histological analysis of key organ tissues (i.e., heart, kidney, lung, and spleen) showed no damages to the tissues and no identifiable immune cells related to implantation. Analysis of complete blood counts and blood chemistry tests also indicated no signs of organ damage or injury, and no changes in the electrolyte and enzyme balance. The system was then implanted in rat brain, showing the possibility to continuously sense cerebral temperature, cerebral oxygen saturation, and neural activity (using a DBR filter coupled with the photodetector and injecting a calcium‐sensitive fluorescent dye, namely, Oregon Green 488 BAPTA 2‐AM) in living animals. Implantation induced minimal inflammatory glial responses (at day 1), which gradually decreased over time to a level comparable to the control group.

The implanted bioresorbable systems discussed in this section clearly point out that the missing tile in bioresorbable opto‐electronics appears nowadays to be efficient and stable implantable power supply sources, which are still in their infancy. In fact, though fully integrated circuits for sensor/transducer driving and readout entirely made of bioresorbable materials were not reported yet, their realization has been plainly envisaged by numerous works on bioresorbable electrical and optical, passive and active components. Eventually, a further challenge to be addressed is related to the operation time of the implanted systems, which is rather short with respect to full dissolution time.

## 6 Open Challenges and Future Directions

**Figure 12:**
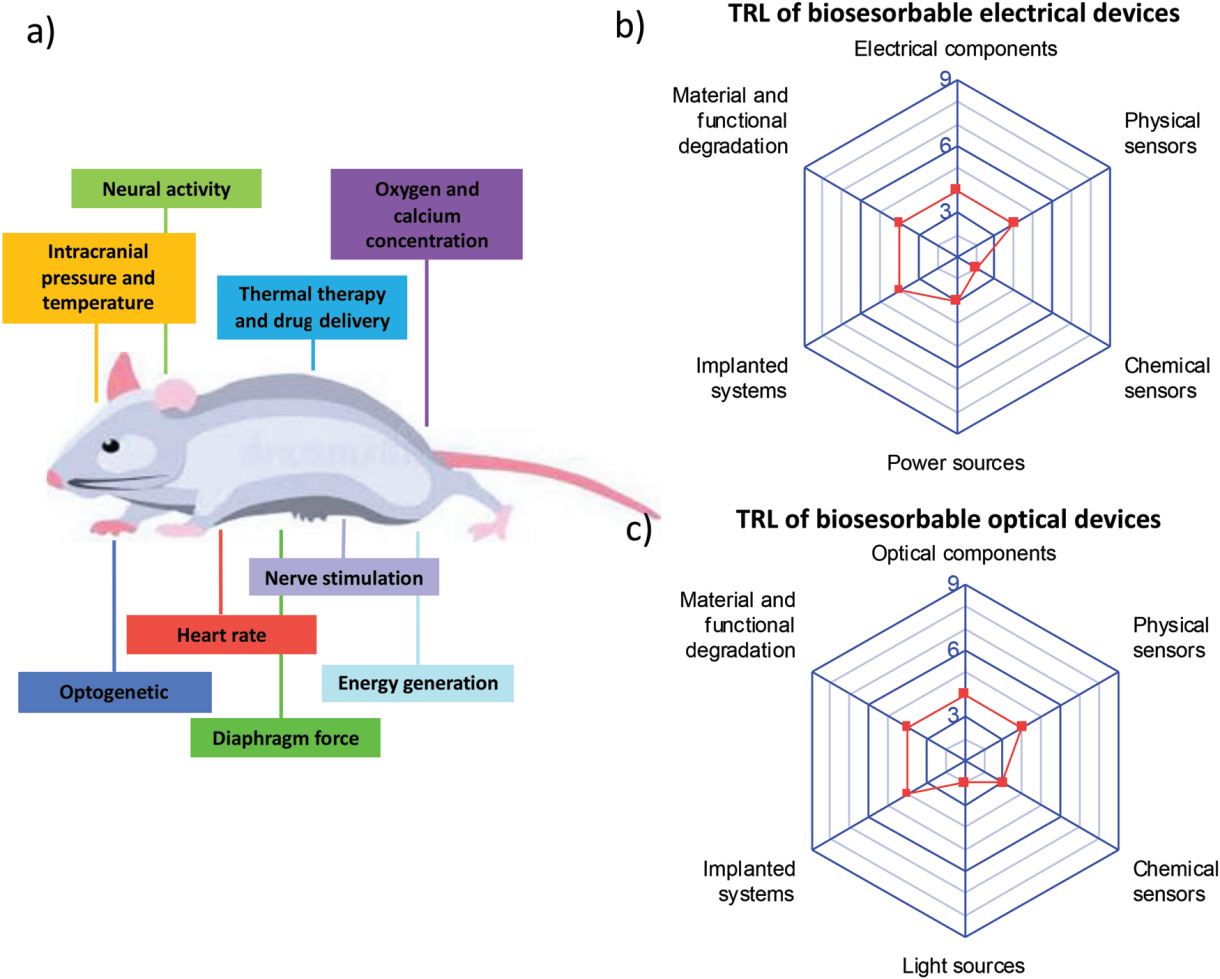
Current picture of bioresorbable devices, systems, and applications. a) Examples of in vivo applications of bioresorbable devices and systems, in animal models. TRL of bioresorbable b) electrical and c) optical devices and systems. Considerations on optical devices are limited to a much lower number of works with respect to electrical devices.

The analysis of the state‐of‐the‐art literature points out that although bioresorbable technology is still in its infancy, a number of important discoveries and applications have been made since the first report on (partially) bioresorbable transistors, in 2009. Remarkably, biocompatibility (though not taking long‐term effects into account), operation, and biodegradability of a variety of both electrical and optical components have been successfully demonstrated in vivo in animal models, which were also employed to provide several proof‐of‐concept demonstrations of potential clinical applications, through the interconnection of a few components into basic sensing systems (**Figure** **12**a and Table 1). In spite of these important achievements, there are challenges that still need to be addressed toward real‐world (and commercial, possibly) applications of such a groundbreaking technology, as clearly highlighted by the technology readiness levels (TRLs)^133^ of bioresorbable components and systems achieved to date, which range from 2 (technology concept and/or application formulated) to 4 (component and/or system validation in laboratory environment). Radar plots in Figure 12b,c summarize (to the best of our knowledge) the TRLs of the different bioresorbable components and systems reported in the scientific literature to date.

The general approach in bioresorbable technology has been based, so far, on the fabrication of a bioresorbable device with a specific functionality, followed by the encapsulation of the device with a protection material. The encapsulation material mainly defines the operation lifetime of the device, by preventing direct contact of the device with biofluids for a prescribed time.

Active and passive, electronic (e.g., transistor, inductor) and optical (e.g., waveguide, photodiode) components and physical sensors (e.g., pressure, temperature) have been advanced at a fast pace, driven by the synergistic interaction of well‐established silicon technology and rapid‐prototyping polymer technologies, which has led to bioresorbable electrical and optical devices of high quality, with good performance, tunable lifetime, and wafer‐scale fabrication (in some cases, at least). Nonetheless, only a few, preliminary attempts to push the technology from components to system level have been reported, the most advanced ones being limited to basic interconnection of sensors with driving or readout components (e.g., use of transistors as switch for multiplexing electrode biopotential measurements, or LCR resonant circuits for wireless transmission with antennas). An open challenge, here, is the fabrication of complex bioresorbable electronic circuits able to promote in situ and in vivo signal amplification, filtering of unwanted signals, signal‐to‐noise ratio improvement, signal modulation for RF transmission. On‐chip fabrication of a bioresorbable operational amplifier (OP‐AMP) composed of tens of transistors connected to resistors and capacitors with simultaneous operation is envisaged to advance the technology, OP‐AMPs being the building‐block of driving, readout, and power management of advanced analog electronic circuits. Further, wireless transmission of signals monitored in situ has been mostly limited to shift of the position of resonance frequency and wavelength of electrical and optical resonant devices, respectively. Electronic circuits enabling analog and/or digital modulation of physicochemical signals recorded in vivo (and demodulation of external analog/digital signals transmitted to impart precise commands/actions to implanted devices) are required to enable an effective wireless data communication and avoid, in turn, wire cables exiting the patient body through skin breaches that are prone to infections.

Besides, chemical sensors and power sources, both electrical and optical, which are essential components of any bioresorbable system designed to operate in vivo, have fallen significantly behind other components.

As to bioresorbable chemical sensors, these have been mostly limited to pH sensing in vitro and oxygen and calcium sensing in vivo. An open challenge, here, is related to stable in vivo operation of these sensors from medium to long time. In fact, differently from active/passive components and physical sensors, in chemical sensors the sensing material must be in direct contact with the biofluid containing the target analytes, so that protection by full encapsulation of these class of sensors with a barrier material is intrinsically not achievable. Research on novel sensing materials and/or functionalization techniques to be used in chemical sensors, with designed specificity to target analytes, stable operation in contact with biofluids, and tunable dissolution on request, is envisaged to advance the field of bioresorbable chemical sensors.

Concerning bioresorbable power sources, all the (primary) batteries reported so far share a low specific energy, with short operation lifetime and rather long full‐degradation time. Water‐based (biocompatible) electrolytes of these bioresorbable batteries lead to enhanced degradation of electrodes (e.g., magnesium‐based anodes), thus limiting the effective energy and the operation lifetime. A challenge, here, is the fabrication of medium to long lifetime high‐performance primary batteries able to operate in vivo (not yet demonstrated) to be used as power supply of implanted devices and systems. In fact, a conventional, external power supply has been commonly used with bioresorbable devices and systems, so far. On the other hand, piezoelectric and triboelectric generators and photovoltaic cells, whose functionality has been already demonstrated in vivo, are unlikely to provide enough continuous energy to power devices and systems over medium to long operation time, being bioresorbable energy storage devices to be connected with not yet available. In this case, the development of a rechargeable (secondary) battery to be used with harvesters would enable energy accumulation and storage, then allowing powering of more complex circuits (e.g., OP‐AMPs) from medium to long times.

Despite optical fibers and photovoltaic and photodetector cells have been assessed in vivo, the development of bioresorbable optical components has been overlooked with respect to the electrical counterpart, with fully bioresorbable LED only recently reported, in fall 2019. Nonetheless, the LED operation has been only reported in vitro with poor performance, both from electrical and optical point of views. Here, an open challenge is connected with the development of light sources, such as LEDs and lasers, able to operate in vivo, to allow bypassing drawbacks related with absorption of tissues and organs in the UV–vis region. In situ light stimulation with implanted LEDs and lasers locally connected with wavelength‐selective photodiodes would allow to avoid connection with externally tethered light sources and signal analyzers.

Eventually, lifetime of all bioresorbable device reported to date ultimately depends on the spontaneous dissolution rate and mechanism of the encapsulating materials in contact with the biofluid, in physiological conditions. Hydrophobic organic polymers (e.g., polyanhydrides) and inorganic materials (i.e., silicon oxides/nitrides) have shown to be excellent encapsulation/barrier layers against water infiltration and to guarantee slow surface erosion for tunable lifetimes. Nonetheless, full degradation of bioresorbable components, once operation lifetime has expired, usually takes a much longer time with respect to operation time, lasting from months to years compared to a few days of device operation. Such a discordance between rapid degradation of performance and slow dissolution of materials in bioresorbable components reported to date represents an open challenge to be addressed in future research toward healthcare commercial applications. Further, being dissolution rate of bioresorbable materials depending on biofluid characteristics (e.g., temperature, pH, and ionic strength), operation lifetime is only roughly preset, especially in vivo. A smart encapsulation layer featuring triggered dissolution upon an external or internal stimulus is envisaged, which allows setting the material lifetime ad‐hoc, depending on medical application needs and patient clinical evolution, by triggering device dissolution. The trigger is required to be specific, to avoid accidental dissolution of the device, and reliable, to ensure dissolution of the device on‐demand. Trigger mechanisms reported so far mostly rely on acceleration of the material dissolution in harsh environments, such as, acidic or basic solutions and high temperatures, and photoinduced depolymerization using light stimuli of suitable wavelength, which requires photosensitizer molecules and photoacid generators. However, none of these approaches has been evaluated in vivo, either in terms of specificity and reliability or biocompatibility. An interesting strategy to be explored is the use of thermoresponsive polymers that change molecular configuration (and hence solubility) when cooled below a tunable threshold temperature (namely, lower critical solution temperature (LCST)), e.g., poly(*N*‐isopropylacrylamide) (PNIPAAM), which has shown good biocompatibility.^134^

## 7 Conclusions

In the last decade an increased research effort has been directed toward the use of biodegradable materials for the fabrication of electrical and optical components designed to operate in vivo for a prescribed time and then dissolve within the body in harmless byproducts, namely, bioresorbable technology. This new trend has germinated from former efforts on biocompatibility and biodegradability investigation of materials used in conventional implantable devices for chronic diseases, perfectly complementing them by providing a means to address short to medium terms medical applications. The challenge is the realization of bioresorbable systems designed to be implanted in the human body and interact with tissues and organs to monitor physiological parameters and/or delivery therapeutic agents, and then dissolved upon request in the body itself generating safe byproducts, without needs for surgical retrieval.

Over the last decade several milestones have been achieved, which have brought such a bioresorbable technology to TRL 4, by demonstrating in vivo operation of a number of devices and systems for some specific clinical applications (e.g., neural activity monitoring, intracranial pressure and temperature recording). On the other hand, many challenges are yet to be addressed toward commercial, real‐world applications of such a technology, such as in vivo operation of bioresorbable batteries, and on‐demand bioresorbability of encapsulation coatings.

Although, in our opinion, real‐world applications on humans will require medium to long terms (5–10 years) research, we do believe that the potential of such a bioresorbable technology is enormous and it can be envisaged that the efforts on such a research topic will increase in the next few years, driven by both clinical and market requests for resorbable devices and systems.

## Conflict of Interest

The authors declare no conflict of interest.
